# DOT1L inhibition is lethal for multiple myeloma due to perturbation of the endoplasmic reticulum stress pathway

**DOI:** 10.18632/oncotarget.27493

**Published:** 2020-03-17

**Authors:** Caroline Dafflon, Swann Gaulis, Louise Barys, Karen Kapur, Vanessa Cornacchione, Lina Schukur, Sebastian Bergling, Elisabetta Traggiai, Selina Jansky, Leon Hellmann, Barbara Schacher Engstler, Grainne Kerr, Antoine de Weck, David A. Ruddy, Ulrike Naumann, Frédéric Stauffer, Christoph Gaul, Ying Lin, Eric Billy, Andreas Weiss, Francesco Hofmann, Moriko Ito, Ralph Tiedt

**Affiliations:** ^1^ Novartis Institutes for BioMedical Research (NIBR) Oncology, Basel, Switzerland; ^2^ NIBR Informatics, Basel, Switzerland; ^3^ NIBR Biologics, Basel, Switzerland; ^4^ NIBR Chemical Biology and Therapeutics, Basel, Switzerland; ^5^ NIBR Oncology, Cambridge, MA, USA; ^6^ NIBR Analytical Sciences and Imaging, Basel, Switzerland; ^7^ NIBR Global Discovery Chemistry, Basel, Switzerland; ^8^ China Novartis Institutes for BioMedical Research, Shanghai, China

**Keywords:** DOT1L, multiple myeloma, epigenetics, histone methylation, unfolded protein response

## Abstract

The histone 3 lysine 79 (H3K79) methyltransferase (HMT) DOT1L is known to play a critical role for growth and survival of *MLL*-rearranged leukemia. Serendipitous observations during high-throughput drug screens indicated that the use of DOT1L inhibitors might be expandable to multiple myeloma (MM). Through pharmacologic and genetic experiments, we could validate that DOT1L is essential for growth and viability of a subset of MM cell lines, in line with a recent report from another team. *In vivo* activity against established MM xenografts was observed with a novel DOT1L inhibitor. In order to understand the molecular mechanism of the dependency in MM, we examined gene expression changes upon DOT1L inhibition in sensitive and insensitive cell lines and discovered that genes belonging to the endoplasmic reticulum (ER) stress pathway and protein synthesis machinery were specifically suppressed in sensitive cells. Whole-genome CRISPR screens in the presence or absence of a DOT1L inhibitor revealed that concomitant targeting of the H3K4me3 methyltransferase SETD1B increases the effect of DOT1L inhibition. Our results provide a strong basis for further investigating DOT1L and SETD1B as targets in MM.

## INTRODUCTION

MM is an aggressive hematologic cancer characterized by the monoclonal expansion of plasma cells secreting high amounts of immunoglobulins. Despite recent progress in therapies, MM still remains an incurable disease and emergence of drug resistance is unfortunately a common feature [1–4]. New treatment options are thus urgently needed. Apart from certain genetic abnormalities, epigenetic mechanisms have been proposed to contribute to the development and maintenance of MM, which gave rise to therapeutic targets like histone deacetylases (HDAC) or EZH2 [5–7].

Due to the high production of immunoglobulins, the protein production machinery in the ER of MM cells is under stress, which is mitigated by a set of pathways known as the unfolded protein response (UPR) in order to ensure survival [8–10]. The sensitivity of MM cells to proteasome inhibitors is thought to be due to perturbation of the hyperactive UPR [11], which may offer some degree of selectivity versus non-cancerous cells [12].

Through pharmacologic and genetic approaches, we found that inhibition of DOT1L, a H3K79 methyltransferase, profoundly reduces viability of a subset of MM cell lines *in vitro* and inhibits growth of established MM xenografts in mice. Our findings confirm a recent study in which DOT1L was suggested as a target in MM [13]. Selective inhibitors of DOT1L with cellular activity (EPZ004777 and SGC0946) [14, 15] have been described, and a further optimized inhibitor (EPZ-5676) [16] has even advanced to clinical trials in *MLL*-rearranged leukemia, where fusion proteins of MLL with a variety of partners recruit DOT1L to a set of genes (e. g. *HOXA9*) that are critical to maintain leukemic cells in an undifferentiated state [14, 16–21]. However, such translocations have not been reported in MM. Instead, we observed that DOT1L inhibition leads to transcriptional reduction of several UPR genes, which may underlie the activity against MM cells. CRISPR screens further revealed that targeting the histone methyltransferase SETD1B concomitantly with DOT1L further enhances this effect on the UPR and increases and accelerates MM cell death. Our data suggest a new approach to treat MM by targeting epigenetic modulators and thereby interfere with the ER stress pathway, which is thought to represent an Achilles’ heel in this type of cancer [8].

## RESULTS

### A subset of MM cell lines is sensitive to DOT1L inhibition

**Figure 1:**
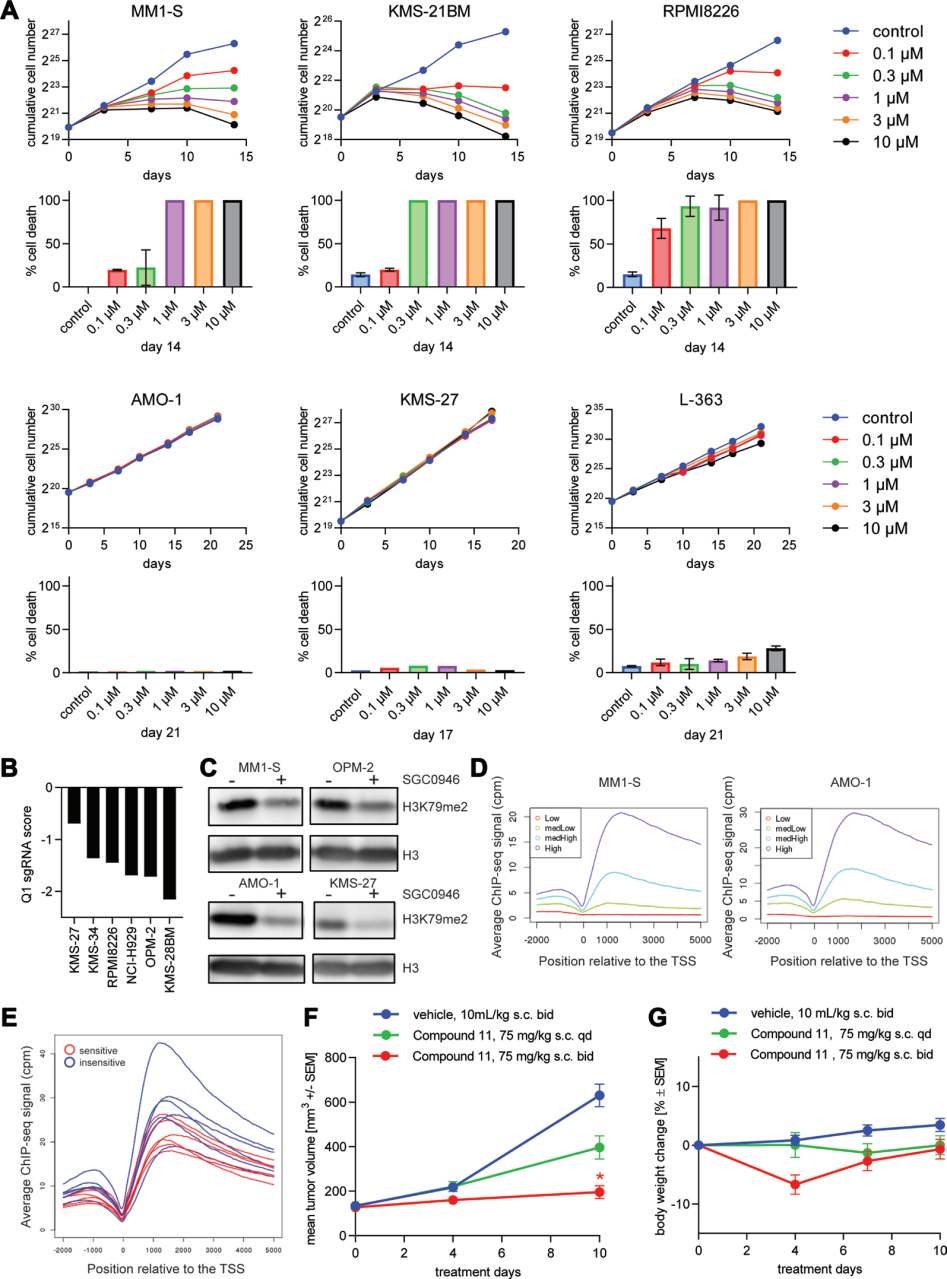
DOT1L inhibition is lethal for a subset of MM cell lines. (**A**) Effect of SGC0946 at different concentrations on the growth and viability of 3 sensitive (upper two rows) and 3 insensitive cell lines (lower two rows) over the course of 14–21 days. The theoretical cumulative number of cells, determined with a Casy TT cell counter, and taking into account dilution factors when passaging the cells, is plotted in the first and third row. Cell numbers may include a fraction of dead or dying cells. Trypan Blue dye exclusion was used to reliably quantify % dead cells at endpoint, which is shown below the respective cumulative cell number plots. (**B**) Bar plot representing the effect of *DOT1L* knockout on viability of MM cell lines after 14 days in context of a whole-genome CRISPR screen. Log2 ratios of sgRNA representation at day 14 compared to the initial library are depicted on the y-axis. First quartile (Q1) values for each cell line were used to summarize the effect of the 10 sgRNAs targeting DOT1L. (**C**) Assessment of global H3K79me2 by western blot in MM1-S, OPM-2 (sensitive cell lines), AMO-1 and KMS-27 (insensitive cell lines). (**D**) H3K79me2 ChIP-seq profiles relative to the TSS for different sets of genes grouped according to their mRNA expression level quantified as counts per million (cpm). (**E**) Averaged ChIP-seq signal of H3K79me2 for 12 MM cell lines related to the TSS (in blue insensitive cells, in red sensitive cells). (**F**) Effect of the DOT1L inhibitor Compound 11 on tumor volume in a MM1-S mouse xenograft model. Female NOD-SCID mice bearing MM1-S-luc subcutaneous xenografts were treated with Compound 11 or vehicle control s. c. at indicated dose-schedules. Values are mean ± SEM, *n* = 8 mice per group. ^*^ *P*-value < 0.05 vs vehicle using Kruskal-Wallis test (Dunn’s post hoc analysis); s. c.: subcutaneous. (**G**) Body weights for *in vivo* experiment shown in (**F**).

Previous studies reported that epigenetic factors are involved in the biology of MM [5]. During pharmacological screens in the Cancer Cell Line Encyclopedia (CCLE) [22], we noticed a modest growth inhibitory effect of DOT1L inhibitors on MM cell lines (data not shown). Given that cell lines were exposed to compounds for only 3 days and DOT1L inhibitors are well documented to act very slowly in *MLL*-rearranged leukemia [14, 16], we validated and extended this observation in a panel of 14 MM cell lines using growth assays with a duration of 2-3 weeks (Figure 1A and Supplementary Figure 1A and 1B). In line with previous findings [13], we observed that growth and viability of a subset of MM cell lines were affected. The majority of MM cell lines could be classified as either sensitive or insensitive to DOT1L inhibition. Proliferation of sensitive cells was reduced by the selective DOT1L inhibitor SGC0946 (Supplementary Table 1) [15] in a dose-dependent manner, and marked cell death was observed. The viability of insensitive cell lines was not, or only modestly, affected by prolonged treatment, although reduced proliferation was observed in some of these cell lines (Supplementary Figure 1B). Importantly, we obtained similar results during a direct comparison of SGC0946 and a second selective but chemically unrelated DOT1L inhibitor, Compound 11 [23] (Supplementary Figure 1C). Differential dependencies on DOT1L were also observed by CRISPR targeting of *DOT1L* in 6 MM cell lines in the context of a whole-genome pooled CRISPR screen [24] (Figure 1B), although segregation into sensitive and insensitive cell lines was less clear. For example, a relatively modest growth reduction seen upon pharmacological DOT1L inhibition in KMS-34 cells (Supplementary Figure 1B) was not distinguished by CRISPR from a much greater pharmacological effect observed in RPMI8226 (Figure 1A).

When measuring global H3K79me2 levels after treatment of MM cells with SGC0946, we observed a reduction of the signal in all MM cell lines (Figure 1C). This result suggests that the drug is active and reaches its target in the insensitive cell lines as well, but H3K79me2 does not seem to be critical for their growth. Moreover, we analyzed the basal H3K79me2 profiles in 6 sensitive and 6 insensitive cell lines by chromatin-immunoprecipitation followed by next generation sequencing (ChIP-seq). The average H3K79me2 levels at the transcription start sites (TSS) of genes correlated with the expression levels of the respective mRNAs as measured by RNA-seq in both sensitive and insensitive cell lines (Figure 1D), confirming that H3K79me2 is a general mark of active transcription [25]. However, H3K79me2 metagene profiles at gene bodies did not discriminate sensitive and insensitive cell lines (Figure 1E). We also compared the occurrence of genomic alterations that are common in MM between sensitive and insensitive lines, but did not observe any significant differences (Supplementary Figure 1D).

So far, only the DOT1L inhibitor EPZ-5676 has been demonstrated to achieve tumor regression in preclinical models. These experiments required rat xenografts of leukemic cell lines that are exceptionally sensitive to DOT1L inhibitors *in vitro* (MV4-11, EOL-1), and continuous intravenous infusion [16, 26]. We recently developed a highly potent DOT1L inhibitor, Compound 11, during a medicinal chemistry program aimed at overcoming the pharmacokinetic limitations of adenosine-containing DOT1L inhibitor such as SGC0946 or EPZ-5676 [23]. Compound 11 can be applied by subcutaneous (s. c.) bolus injection in mice. We thus investigated the effect of DOT1L inhibition in an *in vivo* MM mouse xenograft model. NOD-SCID mice bearing established MM1-S tumors were treated with 75 mg/kg Compound 11 s. c. once or twice daily. Significant tumor growth inhibition was observed with the twice-daily schedule, while a once-daily schedule only led to a moderate effect on tumor growth (Figure 1F). Thus, like in *MLL*-rearranged models, sustained DOT1L inhibition is required for therapeutic efficacy [16]. Despite initial body weight loss in the twice-daily group (Figure 1G), full recovery was seen after 10 days. Efficacy in the MM xenograft was comparable to the anti-tumor effect observed with the same regimen in mice bearing xenografts of the *MLL*-translocated MV4-11 cell line [23]. *In vivo* studies with additional MM xenograft models derived from both sensitive and insensitive cell lines remain to be performed before assessing whether *in vivo* efficacy reproduces the *in vitro* sensitivity pattern. Nevertheless, our *in vitro* and *in vivo* data establish DOT1L as a potential new therapeutic target in MM.

### DOT1L inhibition impairs regulation of ER stress via the key node ATF4

**Figure 2:**
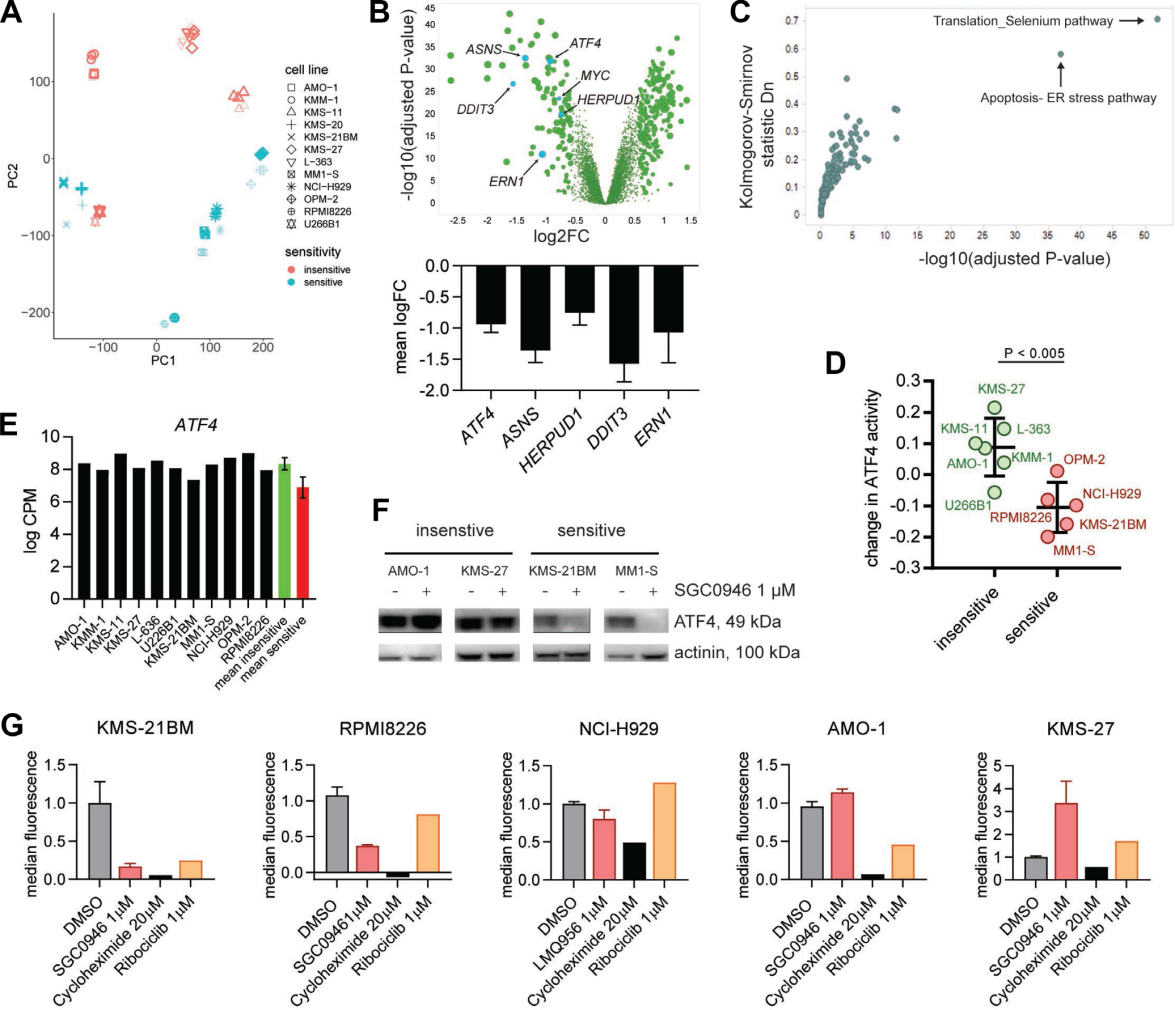
DOT1L inhibition in MM affects the ER stress pathway. (**A**) Principal component (PC) analysis of RNA-seq data from 12 MM cell lines treated with vehicle (DMSO, bold symbols) or 1 μM SGC0946 for 6 days. (**B**) Volcano plot visualizing how sensitive and insensitive cell lines differ with regard to gene expression changes upon SGC0946 treatment (upper panel). The mean of log2-transformed fold changes (logFC) in 6 sensitive cell lines minus the mean logFC in 6 insensitive cell lines is plotted on the x-axis. *P*-values derived from comparison of expression changes in sensitive versus insensitive lines for the respective genes are plotted on the y-axis. Bar graphs showing logFC (mean ± s. d.) for 5 genes related to the ER stress pathway (lower panel). (**C**) Pathway analysis of the genes preferentially downregulated in sensitive versus insensitive cell lines upon DOT1L inhibition. (**D**) Change in activity of the TF ATF4 determined by ISMARA in sensitive and insensitive cell lines upon treatment with 1 μM SGC0946 compared to DMSO. Suppression of ATF4 activity was significantly stronger in sensitive cell lines (*P*-value < 0.005, *t*-test). Mean ± s. d. is indicated. (**E**) Basal level of ATF4 expression in the different MM cell lines. No significant difference between sensitive and insensitive cell lines (*P*- value > 0.05). Mean ± s. d. for red and green bar; cpm: counts per million. (**F**) Assessment of ATF4 by western blot in different sensitive (KSM-21BM and MM1-S) and insensitive cell lines (AMO-1 and KMS-27) treated with either 1 μM SGC0946 or DMSO. (**G**) Quantification of mean fluorescence intensity in HPG+ cells by flow cytometry as indicator of protein synthesis. Cells were pretreated for 7 days with either 1 μM SGC0946 or DMSO or 1 μM ribociclib. A treatment with 20 μM cycloheximide for 8 h was included as control. Bar graphs show the mean ± s. d. normalized to DMSO control, cycloheximide and ribociclib: *n* = 1.

In order to gain insights into the mechanism by which DOT1L inhibitor affects growth and survival of MM cells, we studied gene expression changes following DOT1L inhibition in the previously characterized 6 sensitive and 6 insensitive MM cell lines. We treated these cells for 6 days with SGC0946 followed by RNA sequencing (RNA-seq). This relatively long treatment duration was chosen, because DOT1L inhibitors are known to act slowly [14], potentially linked to the need for histone turnover to remove the H3K79me2 mark [27]. Principal component (PC) analysis revealed tight clustering by cell line identity (Figure 2A). PC2 was able to segregate sensitive and insensitive cell lines into two regions with the exception of U266B1, and the effect of DOT1L inhibition was evident in sensitive cells. Differential gene expression analysis identified several genes that were consistently up- or downregulated in sensitive compared to insensitive cells (Figure 2B). Given the association of H3K79me2 with active transcription, we focused on genes that showed consistent suppression upon treatment among sensitive but not insensitive cell lines (197 transcripts corresponding to 181 genes with *P*- value <10^-10^ and ≥0.7 average log2-fold decrease). Interestingly, genes involved in the ER stress pathway and protein translation, including *ATF4*, *ASNS*, *ERN1* (IRE1α), *DDIT3*, *HERPUD1*, and *MYC* were among these genes (Figure 2B and 2C). Suppression was persistent during continuous treatment for 2 weeks (Supplementary Figure 2A). H3K79me2 basal levels of these DOT1L target genes involved in the ER stress pathway did not differentiate sensitive and insensitive MM lines (Supplementary Figure 2B and data not shown). On a genome-wide level the intensity of the H3K79me2 signal at basal level did not correlate with a differential modulation of gene expression upon DOT1L inhibition (Supplementary Figure 2C).

To further characterize the DOT1L inhibition gene signature, we analyzed which transcription factors (TFs) could explain the observed changes using ISMARA [28]. This computational tool calculates the activity of different TFs from global gene expression profiles and TF binding sites in the regulatory regions of genes. The activity of a TF in a certain context reflects to which extent this specific TF could explain gene expression. Interestingly, this analysis identified ATF4 as a key regulator of genes that are suppressed upon DOT1L inhibition in sensitive cell lines (Figure 2D and Supplementary Table 2). Other transcription factors whose activity was specifically lowered in sensitive cell lines included DDIT3, ATF6 and PRDM1 (Supplementary Table 2). Neither basal mRNA expression of *ATF4* (Figure 2E) nor ISMARA-based basal ATF4 activity (Supplementary Figure 2D) showed a significant difference between sensitive and insensitive cell lines. However, in keeping with the suppression of ATF4 and ATF4 target genes by DOT1L inhibition on the mRNA level, we could detect a reduction of ATF4 protein upon SGC0946 treatment in 4/6 sensitive but none of the insensitive cell lines (Figure 2F and Supplementary Figure 2E).

To identify features that distinguish sensitive from insensitive cells, we also analyzed differential gene expression at baseline. Even with stringent criteria (adjusted *P*-value ≤ 10^-10^, average difference between groups at least 4-fold), we noted a large number of differences: 1557 transcripts for a total of 1089 genes showed higher expression in sensitive cell lines, 637 different transcripts for a total of 271 genes showed lower expression (Supplementary Table 3). Of note, numerous genes belonging to the MHC class II cluster were among the genes with lower expression in sensitive cell lines, whereas many genes with higher expression encode plasma membrane proteins, proteins involved in cell adhesion, and proteins with an immunoglobulin-like fold (Supplementary Table 3). Genes related to regulation of ER stress and protein translation (*ATF4*, *ASNS*, *ERN1, DDIT3*, *HERPUD1*, *MYC, XBP1, EIF2AK3, ATF6*) were not among the genes with differential baseline expression. We also did not observe consistent differences that would distinguish sensitive from insensitive cell lines in terms of basal levels or DOT1L inhibitor-induced changes of the key ER stress regulators IREα (phospho- or total) or phospho-PERK (Supplementary Figure 2F).

Gene expression changes upon DOT1L inhibition suggest a reduction in protein synthesis. To test this directly, cells were treated with the DOT1L inhibitor SGC0946 in the presence of L-homopropargylglycine, which gets incorporated into newly synthesized proteins that can be quantified by flow cytometry after chemoselective ligation of Alexa Fluor 488 azide. Sensitive cell lines synthesized less protein under DOT1L inhibitor treatment compared to insensitive cell lines (Figure 2G). We also included the CDK4/CDK6 inhibitor ribociclib to investigate if cell cycle blockade is generally associated with reduced protein synthesis. Unlike SGC0946, ribociclib significantly diminished proliferation of KMS-27 cells (data not shown), but the protein synthesis levels were comparable to untreated cells, demonstrating that protein synthesis rate is not strictly linked to proliferation.

To further study the impact of ER stress modulation on MM cells, we treated cells with thapsigargin, a known inducer of ER stress [10]. Unexpectedly, we observed that this treatment, which has opposing effects to DOT1L inhibition on several ER stress-related genes [29, 30], still segregated MM cells into the same sensitivity groups (Supplementary Figure 2G). However, the combination of thapsigargin with SGC0946 did not show antagonistic effects. These results suggest that some MM cell lines are particularly sensitive to perturbations of the UPR, while other cell lines are not.

### DOT1L inhibition decreases the number of antibody-secreting cells in sensitive MM cell lines

We also investigated the effect of DOT1L on antibody production. Immunoglobulins are composed of two heavy chain subunits and either two kappa or two lambda light chain subunits. In MM, the type of light chain influences clinical prognosis, lambda being worse than kappa, and some MM cases produce only free light chains [31]. Using flow cytometry, we first characterized the type of light chain that is produced in several MM cell lines (Supplementary Figure 3A). Combined with published data for additional MM cell lines, we observed that the fraction of cell lines producing lambda light chains was higher among sensitive cells, although this difference did not reach statistical significance (Fisher’s exact test, *P*-value < 0.17) (Supplementary Figure 3B).

**Figure 3:**
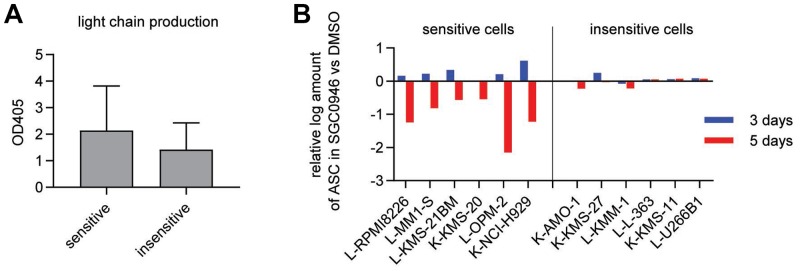
Number of antibody-secreting cells is reduced in sensitive cell lines. (**A**) Light chain secretion quantified by ELISA in supernatants from 6 sensitive (MM-1-S, OPM-2, KMS-21BM, RPMI8826, NCI-H929 and KMS-20) and 6 insensitive cell lines (AMO-1, KMM-1, KMS-11, U266B1, KMS-27 and L-363) without DOT1L inhibitor treatment (DMSO only). OD 405: optical density at 405 nm. Bar graphs show the mean ± s. d. (**B**) Enumeration of antibody-secreting cells (ASCs) by ELISPOT in 6 sensitive and 6 insensitive cell lines treated with either 1 μM SGC0946 or DMSO for the indicated durations. The y-axis represents the log2 ratio of ASCs in the SGC0946-treated compared to the DMSO group. L: lambda chain, K: kappa chain.

To quantify the antibody production in MM cell lines, we measured secreted light chains by ELISA assays. No significant difference between sensitive and insensitive cell lines was observed in absence of DOT1L inhibitor (Figure 3A), and the effects in presence of DOT1L inhibitor were difficult to quantify on a per-cell basis due to the concomitant effect on cell growth. We thus performed ELISPOT assays to quantify the number of antibody-secreting cells (ASCs) and found that the numbers of ASCs were reduced specifically in the sensitive group upon treatment (Figure 3B and Supplementary Figure 3C). However, the rate of light chain secretion per ASC was not diminished by DOT1L inhibition (Supplementary Figure 3D). In line with this observation, ELISPOT assays did not reveal any apparent difference in spot size between DOT1L inhibitor-treated and control conditions (Supplementary Figure 3E) and surface immunoglobulin expression was not affected by DOT1L inhibition either (Supplementary Figure 3A).

### Loss of SETD1B sensitizes MM cells to DOT1L inhibition

**Figure 4:**
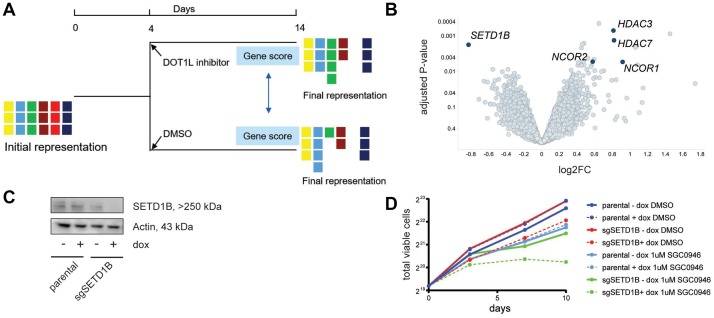
Whole-genome CRISPR screen with or without DOT1L inhibition indicates sensitization by *SETD1B* inactivation. (**A**) Schematic of the screening strategy. MM cells were infected with a pooled lentiviral sgRNA library, selected with puromycin and then divided into two populations: DMSO control and compound treatment (1 μM SGC0946). After 10 days, genomic DNA was isolated from cells, sgRNA sequences were PCR-amplified and abundance of individual sgRNAs was quantified by deep-sequencing. (**B**) Volcano plot of sensitizers and rescuers in the 4 sensitive cell lines. Each point represents a gene, the x-axis indicates the mean log2-fold changes in sgRNA representation across the 4 cell lines when comparing SGC0946 treatment to DMSO, and the y-axis represents the significance of the log-fold changes (-log10 of *P* value). (**C**) Assessment of *SETD1B* knockout efficiency by western blot in RMPI8226 Cas9 cells upon activation of an inducible sgRNA targeting *SETD1B* with doxycycline (dox, 100 ng/ml) for 3 days. (**D**) Effect of *SETD1B* knockout on growth of RPMI8226 Cas9 cells in the presence or absence of SGC0946. Expression of sgSETD1B was induced with dox as before. Parental RPMI8226 Cas9 cells without sgRNA are shown as control.

We decided to conduct whole-genome CRISPR screens in presence or absence of a DOT1L inhibitor in a set of MM cell lines, reasoning that (1) identification of sensitizers (i. e. genes whose inactivation further enhances the effect on cell viability) could have practical therapeutic value and (2) identification of rescuers (i. e. genes whose inactivation restores viability in presence of DOT1L inhibitor) could yield mechanistic insights into the DOT1L dependency of some MM cell lines. Four sensitive, one intermediate, and one insensitive cell line were screened. Following transduction with the sgRNA libraries and selection, cells were treated with either DMSO or the DOT1L inhibitor Compound 11 (Figure 4A). 14 days after transduction, the abundance of each sgRNA relative to its representation in the initial plasmid pool was quantified by deep sequencing and fold changes of each gene between treatment and DMSO were calculated. Across all sensitive cell lines, depletion of sgRNAs targeting *SETD1B* in the DOT1L inhibitor arm relative to the control arm was the most pronounced sensitizing effect (Figure 4B). Among the rescuers in these cell lines, we observed components of nuclear co-repressor complexes.

*SETD1B* is a member of the *MLL* H3K4me3 methyltransferase family [32]. To validate the sensitization effect, we transduced RPMI8226 Cas9 cells with a doxycycline (dox)-inducible *SETD1B* sgRNA construct that led to SETD1B protein loss upon induction (Figure 4C). In keeping with the screening data, a stronger and more rapid reduction of the proliferation rate of RPMI8226 Cas9 cells upon SGC0946 treatment was observed when *SETD1B* KO was induced simultaneously (Figure 4D).

**Figure 5:**
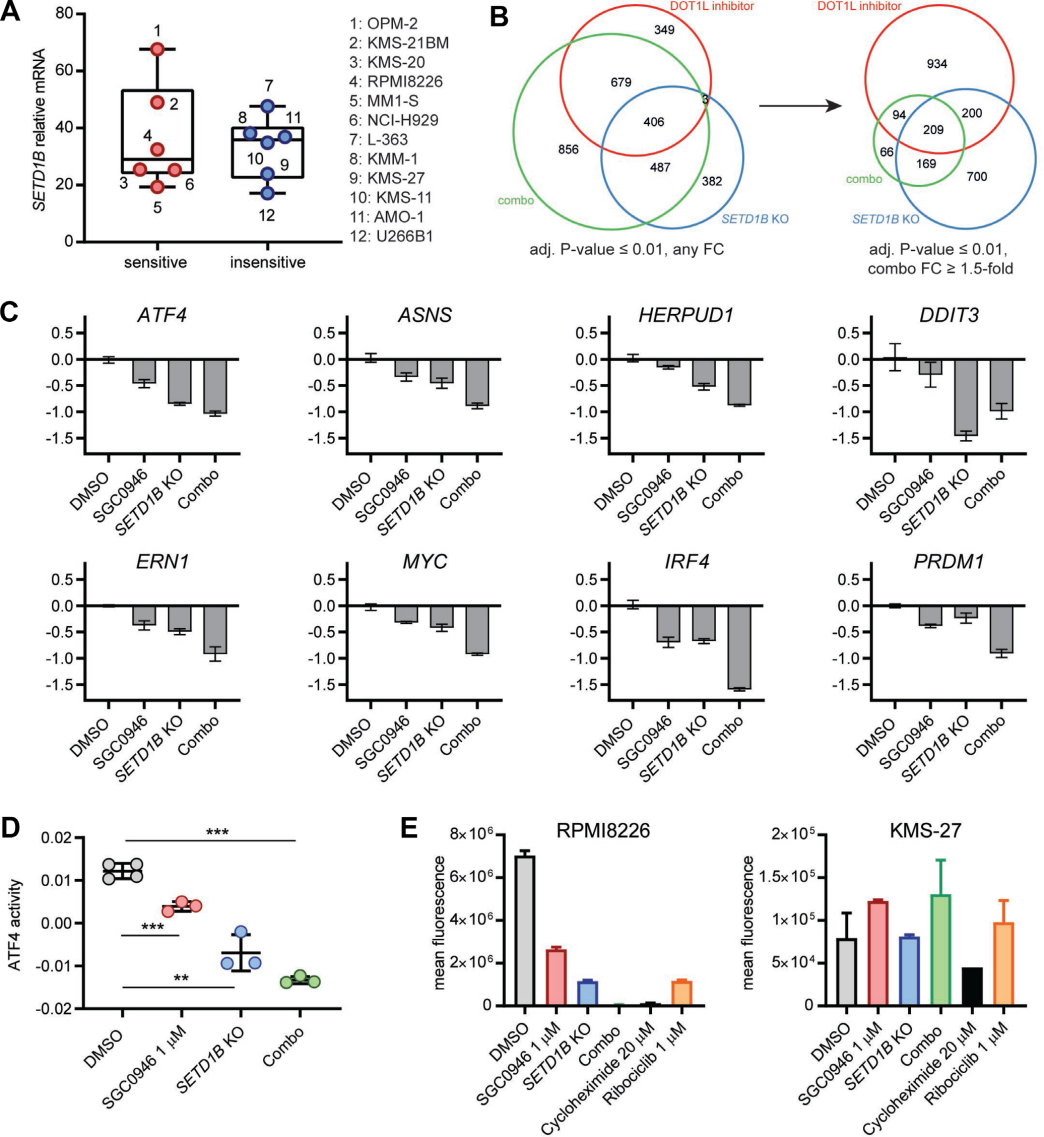
Combined inactivation of DOT1L and *SETD1B* in MM affects the ER stress pathway. (**A**) Box-and-whiskers plot showing expression of *SETD1B* mRNA as measured by RNA-seq at basal level in MM cells. No significant difference between sensitive and insensitive cell lines (*P*-value > 0.05). (**B**) RPMI8226 Cas9 cells transduced with dox-inducible sgSETD1B were treated for 3 days with either DMSO, 1 μM SGC0946, dox (100 ng/ml) or a combination of both, gene expression changes were then measured by RNA-seq. Area-proportional Venn diagrams show the overlap between significantly downregulated genes (each experimental condition compared to DMSO, adjusted *P*-value ≤ 0.01). Left: any fold changes, right: combination group further filtered to at least 1.5-fold reduced genes. (**C**) Expression of individual ER stress genes (*ATF4*, *ASNS*, *HERPUD1*, *DDIT3* and *ERN1*) as well as other key regulators of MM biology (*MYC*, *IRF4*, *PRDM1*) from the same experiment as in (B); tpm: transcripts per million. (**D**) Activity of the TF ATF4 by ISMARA using the same RNA-seq data as in (B). All *P*-values were obtained from unpaired two-sided *t*-tests: ^***^ *P*-value < 0.001, ^**^ *P*-value < 0.01. (**E**) Quantification of protein synthesis by flow cytometry. RPMI8226 Cas9 and KMS-27 Cas9 (both transduced with inducible sgSETD1B) were treated for 7 days with either DMSO, 1 μM SGC0946, 100 ng/ml dox, a combination of SGC0946 and dox, or 1 μM ribociclib. As a control, cells were treated with 20 μM cycloheximide for 8 h. Bar graphs show the mean fluorescence of HPG+ cells ± s. d.

We did not observe a systematic difference in *SETD1B* basal expression between cell lines (Figure 5A). To decipher the mechanism by which DOT1L and SETD1B interact functionally, we studied gene expression changes in RPMI8226 following DOT1L inhibition, dox-induced *SETD1B* KO, or a combination thereof for 3 days. Focusing on the downregulated genes, we noted that the largest number of significant changes (adjusted *P*-value ≤ 0.01) occurred in the combination group, overlapping substantially with each of the two other experimental conditions (Figure 5B, left). The overlap between DOT1L inhibition and *SETD1B* KO induced changes was less pronounced. Considering only the genes that were reduced at least 1.5-fold by the combination, we saw an even greater overlap with either of the two other conditions, and 209 of those genes were significantly changed in all conditions (Figure 5B, right). Interestingly, these 209 genes included previously identified ER stress-related DOT1L target genes, suppression of which was reinforced upon combined *SETD1B* and DOT1L targeting with the exception of *DDIT3* (Figure 5C and Supplementary Table 4). Moreover, other critical regulators of MM biology, like *MYC*, *IRF4*, or *PRDM1*, as well as *BCL2* and *TNFRSF17 (BCMA)* [33–35], were also highly downregulated by combination treatment (Figure 5C). GSEA (gene set enrichment analysis, Supplementary Figure 4A) and gene set overlap analyses (Supplementary Table 4) consistently revealed that the combination suppressed genes with a role in translation, the UPR and the ER stress pathway. While not meeting the fold-change criterion above, expression of the immunoglobulin regulator XBP1 was also significantly reduced in the combination group (Supplementary Table 4). Quantification of the activity of the TF ATF4 by ISMARA unveiled a reduction upon inactivation of either DOT1L or SETD1B, which was even more pronounced after combined inactivation (Figure 5D). Reduced activity was also detected for DDIT3 and XBP1 as well as MYC and PRDM1 (Supplementary Figure 4B), consistent with the suppression of their mRNAs upon combined DOT1L and *SETD1B* targeting. Furthermore, protein synthesis in RPMI8226 cells was reduced in a combinatorial manner as well (Figure 5E). Protein synthesis was not reduced by equivalent perturbations in the insensitive cell line KMS-27. These results suggest that DOT1L and SETD1B are both critical for maintaining protein synthesis, which is strongly reduced when both are targeted concomitantly.

### DOT1L target loci are not associated with abnormal H3K79me2 and H3K4me3 in MM cells

**Figure 6:**
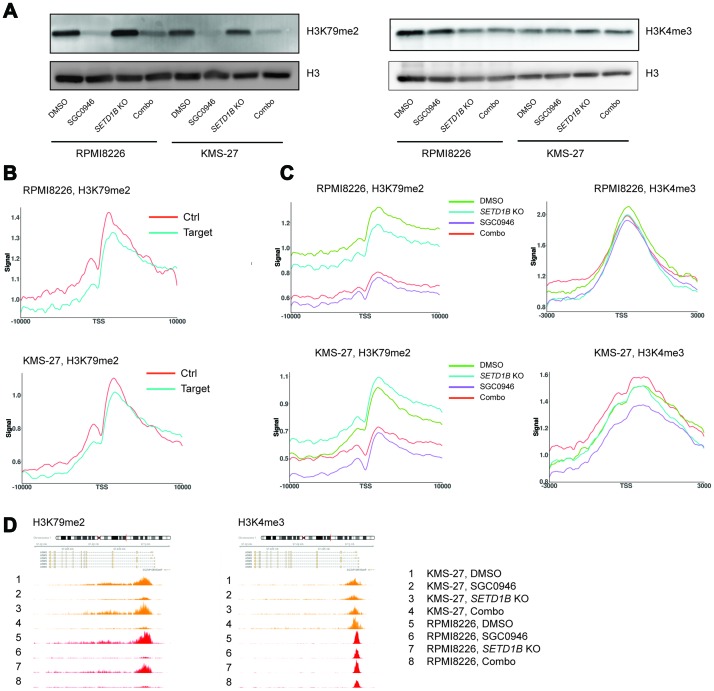
Genome-wide profiles of H3K79me2 and H3K4me3 upon perturbation of DOT1L and *SETD1B*. (**A**) Assessment of H3K79me2 and H3K4me3 by western blot in RPMI8226 Cas9 and KMS-27 Cas9 cells containing inducible sgSETD1B, treated for 4 days with either DMSO, 1 μM SGC0946, 100 ng/ml dox or a combination of SGC0946 and dox. (**B**) ChIP-seq H3K79me2 metagene profiles around the TSS of DOT1L target genes compared to control genes with similar expression levels in RPMI8226 and KMS-27 Cas9 cells at basal level (DMSO treatment). (**C**) ChIP-seq H3K79me2 and H3K4me3 metagene profiles around the TSS of DOT1L target genes in RPMI8226 and KMS-27 Cas9 cells containing inducible sgSETD1B, treated as in (A). (**D**) ChIP-seq tracks representing signal for H3K79me2 and H3K4me3 around *ASNS* gene in RPMI8226 Cas9 cells (orange) and KM-S27 Cas9 cells (red), treated as in (B).

To assess how perturbation of DOT1L and/or SETD1B changes the corresponding histone marks H3K79me2 and H3K4me3 across the genome, RPMI8226 Cas9 (sensitive) and KMS-27 Cas9 (insensitive) cells containing inducible sgSETD1B were treated with either DMSO, 1 μM SGC0946, dox (to induce *SETD1B* KO) or a combination of both. Genome-wide H3K79me2 and H3K4me3 profiles were then acquired by ChIP-seq (Supplementary Figure 5A). Of note, western blots did not reveal any differential suppression of H3K79me2 on the global level or any noticeable suppression of H3K4me3 (Figure 6A). Therefore, we initially focused on histone mark profiles on the previously identified DOT1L target genes. To normalize the ChIP-seq data, we added 5% of Drosophila spike-in chromatin as a reference [36]. This method had been shown to be critical to detect relevant epigenomic changes upon DOT1L inhibitor treatment, where global H3K79me2 changes are expected. For the purpose of our analysis, DOT1L target genes were defined as genes with an average log2FC > 0.68 and a *P*-value < 10^-10^ (Figure 2B). In *MLL-AF9* rearranged leukemic cells, H3K79me2 levels at target genes of the MLL fusion protein were reported to be on average higher and more spread out than at non-target genes [37]. We searched for analogous abnormal H3K79me2 profiles in MM at the DOT1L target genes and created a control set of genes that showed the same basal distribution of mRNA expressions as the DOT1L target genes. The average level and spread of H3K79me2 relative to TSS in the control set was similar to the DOT1L target genes (Figure 6B), thus arguing against an H3K79me2 epigenetic anomaly at DOT1L target loci. Next, we assessed the H3K79me2 profile at DOT1L target genes under the different treatment conditions. Interestingly, in sensitive cells H3K79me2 levels were equally reduced at DOT1L target genes and control genes, both upon DOT1L inhibition alone and in combination with *SETD1B* targeting (Figure 6C). Even though the expression of DOT1L target genes was not changed following DOT1L inhibition in insensitive cells, H3K79me2 was strongly reduced (Figure 6C). We then performed the same analyses for ATF4 target genes, since ATF4 was identified as a possible key mediator of the DOT1L signature. Similar results were obtained as for DOT1L target genes (Supplementary Figure 5B). Remarkably, no major differences were noted between sensitive and insensitive cells when we analyzed the H3K79me2 and H3K4me3 profiles upon DOT1L and/or *SETD1B* perturbation (Figure 6C, 6D and Supplementary Figure 5C). Overall, these results suggest that the dependency of a subset of MM cell on DOT1L is complex and not readily explainable by either basal or treatment-modulated H3K79me2 and H3K4me3 levels.

## DISCUSSION

DOT1L is an established preclinical target in *MLL*-rearranged leukemia [25], and some degree of clinical activity was observed with the first candidate that was tested in leukemia trials [38]. Here, we describe the discovery and characterization of a novel therapeutic opportunity for DOT1L inhibitors in MM. Interestingly, we found a distinct pattern of sensitivity across MM cell lines that was observed using two DOT1L inhibitors with very different chemical structures. The most consistent transcriptional effect of DOT1L inhibition on sensitive MM cells was a suppression of UPR genes, which may largely be mediated by DOT1L-induced suppression of the transcription factor ATF4. One consequence of these transcriptional changes was a reduction of global protein synthesis, concomitant with lower production of immunoglobulins. Whether these effects are causal for the observed loss of viability, and by which mechanism, needs to be investigated in future studies. Of note, perturbation of the ER stress level using thapsigargin also affected growth and viability of the same MM cell lines as DOT1L inhibition, suggesting a general sensitivity towards changes in ER stress / UPR in these cells [39]. We also found numerous genes with lower or higher expression in sensitive MM cell lines at baseline, which may serve to predict sensitivity. However, there was no apparent link between baseline differences and gene expression modulation upon DOT1L inhibition.

When we conducted genome-wide CRISPR screens in the presence or absence of a DOT1L inhibitor in MM cell lines, the H3K4me3 methyltransferase SETD1B emerged as a robust sensitizer. We could validate that targeting *SETD1B* reinforces the effect of DOT1L inhibition, notably the suppression of some genes of the UPR pathway. Moreover, combined targeting in RPMI8226 cells led to suppression of several other genes that are linked to MM biology and could plausibly contribute to the observed growth effect. These include *MYC* [40] and the *IRF4* axis (*IRF4*, *PRDM1*) [41]. In summary, DOT1L and SETD1B seem to be critical for maintaining the expression of several genes that are key to the biology of MM.

In *MLL*-rearranged leukemia, DOT1L is thought to maintain expression of leukemia stem cell genes through recruitment via MLL fusion proteins to these gene loci, leading to higher H3K79me2 levels. To investigate whether DOT1L exerted such a locus-specific effects on its target genes or on ATF4 target genes in MM, we conducted ChIP-seq experiments. These studies revealed no distinct H3K79me2 profile at genes that are modulated upon DOT1L inhibition. Moreover, reduction of H3K79me2 at those modulated genes was comparable in sensitive and insensitive cells, although mRNA expression was not modulated in the latter. Thus, we did not find distinguishing behavior of MM cells on the chromatin level. Remarkably, *SETD1B* knockout led to very few differential H3K4me3 peaks in both sensitive and insensitive cells (data not shown) despite SETD1B being a H3K4 methyltransferase. This could well be a consequence of redundancy among H3K4 methyltransferases, although it raises the question what mediates the observed sensitization and gene expression modulation.

In summary, our discovery indicates a novel opportunity for DOT1L inhibitors in cancer treatment. Concomitant targeting of *SETD1B* enhances phenotypic and transcriptional effects of DOT1L inhibition in sensitive MM cell lines, which may be an additional therapeutic angle. To our knowledge, no selective SETD1B inhibitors have been described yet, and our data questions whether its methyltransferase activity is critical in the MM context. Additional work is needed to address this question and guide drug discovery.

## MATERIALS AND METHODS

For additional information, see Supplementary Materials and Methods.

### Whole-genome CRISPR screen and analysis

#### Screening approach

A whole-genome CRISPR screen was performed in 6 MM cell lines (KMS-27, KMS-34, OPM-2, RPMI8226, KMS-28BM and NCI-H929), in which each gene was targeted by 10 sgRNAs. Experimental procedures were detailed previously [24, 42]. Following transduction and selection with puromycin, treatment of cells with either DMSO or SGC0946 was initiated on day 4. Abundance of each sgRNA in cells on day 14 relative to its representation in the initial plasmid pool was then assessed by next-generation sequencing (NGS).

### Quantification and statistical analysis

The drop-out value for each sgRNA was calculated using the Bioconductor R package EdgeR [43]. The plasmid (representing day 0) and sample raw counts per sgRNA were normalized in pairs using the Trimmed Mean of M-values (TMM) normalization. To measure the viability effect after 14 days of growth, the edgeR negative binomial model was fitted to obtain the log-fold change (logFC) of counts between the sample and plasmid (the parameters used are common dispersion = 0.2 and prior count = 12). This was performed for each sample-plasmid pair to obtain a logFC per sgRNA per cell line. The logFC were then normalized using quantile normalization to obtain a sgRNA level sensitivity score. As a summarized gene level score, we used the Q1 logFC of the 10 sgRNAs targeting it.

To identify statistically significant sensitizers and rescuers between the treatment and DMSO groups of cell lines at day 14, for each sample a gene-summarized value was first calculated as the median count of sgRNAs targeting it. The edgeR package was then used to fit negative binomial generalized log-linear model to the summarized count data and to conduct gene-wise statistical tests for the contrast ~Treatment-DMSO.

### H3K79me2 and H3K4me3 ChIP-seq

Details can be found in the Supplementary Material and Methods. Briefly, cells were crosslinked with 1% formaldehyde and lysed in SDS buffer. DNA was fragmented by sonication. ChIPs for H3K79me2 and H3K4me3 were then performed and eluted DNA fragments were subjected to NGS.

### Protein synthesis assay

For detection of newly synthetized proteins, Click-iT reagents and buffers were used (Invitrogen, #C10428). MM cells were treated during 7 days either with DMSO, 1 μM of SGC0946 or 1 μM ribociclib. As a control, cells were treated with 20 μM cycloheximide for 8 h. For analysis of newly synthetized proteins, cells were then washed and grown in methionine-free media for 30 min containing Click-iT HPG (50 μM). After incubation, cells were washed with PBS and then fixed with 3.7% formaldehyde, washed with 3% BSA and then 0.5% Triton-X-100 was added to the cells. For the detection of Click-iT HPG, Click-iT reaction cocktail containing the AlexaFluor 488 azide. The analysis of nascent protein synthesis was then performed by flow-activated cell sorter (FACS) (Cytoflex S, Beckman Coulter).

## SUPPLEMENTARY MATERIALS






