# Supplementary material for: DOT1L inhibition is lethal for multiple myeloma due to perturbation of the endoplasmic reticulum stress pathway
Source: Oncotarget. 2020 Mar 17;11(11):956–68. doi: 10.18632/oncotarget.27493 (PMC7082114; doi:10.18632/oncotarget.27493)
Supplement: Supplementary file 1 [file oncotarget-11-956-s001.pdf]

DOT1L inhibition is lethal for multiple myeloma due to perturbation of the endoplasmic reticulum stress pathway

SUPPLEMENTARY MATERIALS

Cell lines and reagents

All MM cell lines were obtained from the CCLE [1] and maintained under standard conditions (37° C, 5% CO2). Cells were grown in RPMI1640 (Gibco, #61870-010) + 20% Fetal Calf Serum (FCS) +10 mM HEPES + 1 mM Sodium Pyruvate. The following reagents were purchased: SGC0946 (DOT1L inhibitor, Sigma-Aldrich, SML 1167- 5MG)[2] and thapsigargin (Sigma-Aldrich, T9033). *Drosophila* S2 cells (Thermo Fisher, # R690-07) were cultured in Insect -XPRESS medium (Lonza, # 12-730F) and used for normalization in the ChIP-seq experiment.

Targeting of *SETD1B* by CRISPR

To knockout *SETD1B,* different inducible sgRNA constructs were cloned into a lentiviral vector (pNGx_LV_g016; Cellecta) with a tetracycline-inducible U6 promoter, and the sgRNA with the most efficient knockout (sg_3) was selected with the target sequence (sense) CATGTGCAAGAAGTATGGGG. Infected pools of RPMI8226 and KMS-27 cells were established and knockout was induced by addition of 100 ng/ml doxycycline. Knockout efficiency was verified by western blot using a SETD1B antibody (Abcam, #ab113984). The immunoblot was probed with an actin antibody (Millipore, #MAB1501 clone C4) for normalization. Detection was performed with a western blotting detection kit (SuperSignal West Femto Maximum Sensitivity Substrate kit, Thermo Fisher).

Whole transcriptome analysis by RNA-seq

RNA-seq of MM cell lines and analysis

12 cell lines (MM1-S, KMS-20, KMS-21BM, OPM-2, RPMI8226, NCI-H929, AMO-1, KMM-1, KMS-11, KMS-27, U266B1, L-363) were treated for 6 days with DMSO or 1 µM of the DOT1L inhibitor SGC0946 in biological triplicates. Total RNA was extracted using the RNeasy Plus Mini Kit (Qiagen, #74136) according to the manufacturer's instructions and submitted to RNA sequencing for quantification of transcript expression. Raw sequencing data was aligned to the human genome (hg19 assembly) using the EQP software [3]. The raw gene-level counts were normalized with the Voom procedure [4] and differential expression analysis was conducted with the Limma R/Bioconductor software [5]. Each SGC0946-treated cell line was contrasted to its respective DMSO baseline and the treated SGC0946-sensitive cell lines were also contrasted to the treated insensitive ones upon DMSO baseline subtraction. Pathway analysis of the RNA-seq data was described previously [6]. For the purpose of gene set enrichment analysis, the differentially expressed genes, obtained as described above by contrasting the SGC0946-treated sensitive to insensitive cell lines, were ranked in descending order according to the fold change signed minus log10 *P*-value of the Limma statistical test. Enrichment of the gene sets extracted from Metacore process network database was assessed with the Kolmogorov–Smirnov (K-S) test. The gene sets significantly over-represented in the SGC0946-treated sensitive or insensitive cell lines were selected by plotting the K-S statistics Dn over the minus log10 P-value of the K-S test.

RNA-seq of RPMI8226 Cas9 with inducible sgSETD1B and analysis

RPMI8226 Cas9 cells (with an inducible sgSETD1B expressing vector) were pretreated during 3 days with doxycycline (100 ng/ml) or not. Cells were then treated with either DMSO, 1 µM SGC0946, doxycycline (100 ng/ml), or combination of doxycycline and 1 µM SGC0946 for another 3 days, followed by total RNA extraction using the RNeasy Plus Mini Kit (Qiagen, #74136) according to the manufacturer's instructions. Illumina-compatible libraries for RNA-sequencing were generated by using the Illumina TruSeq Stranded Total RNA Sample Preparation Kit (including Ribo-Zero Gold rRNA depletion) and custom Library-Adapters ordered from IDT. 12 cycles of PCR were applied for library enrichment. Pathway analysis of the RNA-seq data was described previously [6].Gene set enrichment analyses (GSEAs) [7] were performed on pre-ranked gene lists using the gene list analysis module of the GSEA desktop java version 2.01.12.

ISMARA analysis

ISMARA analysis was performed using the online version of this tool [8]. Activity of a transcription factor in a sample defines how much expression (log2 transformed)

of a promoter in this sample changes per 1 binding site of this TF in proximity of this promoter.

H3K79me2 and H3K4me3 ChIP-seq and bioinformatic analysis

ChIP-seq

RPMI8226 Cas9 sgSETD1B and KMS-27 Cas9 sgSETD1B cells were treated as described in the Results section and then cross-linked for 10 min in 1% formaldehyde at room temperature. The reaction was stopped by adding 0.125 M glycine. Cells were lysed in SDS buffer (100 mM NaCl, 50 mM Tris-Cl at pH 8.0, 5 mM EDTA, 0.02% NaN3, 0.5% SDS) and then harvested in ChIP buffer (SDS buffer mixed 1:1 with Triton Dilution buffer- 100 mM Tris-Cl at pH 8.6, 100 mM NaCl, 5 mM EDTA pH 8.0, 0.02% NaN3, 5% Triton X-100). Samples were sonicated using an Active Motif Epishear to obtain fragments of average 200–500 bp in size. *Drosophila* S2 cells were also collected with the same method. S2 chromatin were then added to human chromatin (10 µg of total chromatin, 5% Drosophila chromatin, 95% human chromatin). Appropriate amounts of chromatin were incubated overnight with the specific antibodies listed below. Immunoprecipitated chromatin complexes were recovered on Protein-G beads (Thermo Fisher, #10003D). After multiple washes DNA was recovered by reverse crosslinking and purification using the QIAquick PCR purification kit (Qiagen, # 28106). ChIP-seq of the 12 MM cell lines without treatment (Figure 1) was performed with the same protocol except that a COVARIAS S2x sonicator was used and that the different buffers were purchased from Millipore (SDS lysis buffer, Millipore, #20-163; ChIP buffer, Millipore, #20-153). Illumina-compatible libraries for ChIP-sequencing were generated by using the NuGEN Ovation Ultralow System V2 1-96 (with A-tailing) Kit (Nugen, Part: 9107-96). 16 cycles of PCR were applied for library enrichment. The following antibodies were used for the ChIP-seq of the 12 MM cell lines: H3K79me2: rabbit (Abcam, #ab3594); H3K4me3: rabbit (Millipore, # 04-745); IgG: rabbit (CST, #2729). The following antibodies were used for the ChIP-seq with RPMI8226 and KMS-27 cells: H3K79me2: rabbit (CST, #5427); H3K4me3: rabbit (Millipore, # 07-473); IgG: rabbit (Sigma, #15006).

Bioinformatic analysis, 12 MM cell lines

For each processed sample, the raw H3K79me2 ChIP-seq sequencing reads were mapped to the human genome (hg19 assembly) with the Bowtie2 software. The sequencing coverage was calculated over genome from the BAM output of the alignment and displayed as a BigWig file in the UCSC genome browser. H3K79me2 positive DNA regions were identified from ChIP-seq data by calling peaks with MACS2.0.10. Differential gene expression was merged with ChIP-seq data in the following way: the consensus ChIP-seq peaks over the 6 sensitive cell lines were computed with the DiffBind 1.12.3 Bioconductor package (with a minimum overlap of peaks in r 4cell lines), and as a measure of ChIP-seq signal for each peak and sensitive cell line RPKM values were calculated with DiffBind. A global composite ChIP-seq signal score was calculated for each peak by summing over the individual cell line ChIP-seq signals. Gene-centric differential expression data (treated SGC0946-sensitive to insensitive cell lines contrast) were merged with the peak-centric ChIP-seq signal by genomic region proximity using the annotate function of the ChIPpeakAnno 3.4.4 Bioconductor package. Metagene profiles were generated as follows: for every human protein coding gene of the ENSEMBL 73 database and per cell line, the base level coverage of ChiP-seq reads was calculated in a –2kb +3kb window centered at gene transcription start sites (TSS) and expressed as counts-per-million reads (CPM). Per cell line the base position coverage averages were calculated over genes overlapping with ChIP-seq sensitive / insensitive consensus peak sets (obtained as described above) and plotted as smoothed lines (smoothing factor 100 bases). All data were calculated with standard packages from R-3.1.3 and associated Bioconductor release.

Bioinformatic analysis, second ChIP-seq experiment with RPMI8226 and KMS-27 cells

Histone ChIP-seq aligment was performed using the AQUAS pipeline (https://github.com/kundajelab/chipseq_pipeline). Drosophila normalization was performed by computing the reference-adjusted read per million (RPM) [9]. Each set of target genes was compared to a set of control genes, where matching was performed on the gene expression level and gene width. Where log-fold changes were available, control genes were required to show absolute logFC < 0.5. Differential binding analysis was performed using DESeq2 with normalization factors. For each histone mark a set of consensus peaks occurring in at least 3 samples were used for downstream analysis. Statistical comparisons of metagene profiles were performed using t-tests.

In vitro growth curves

Cells were seeded into 24-well plates at a density of 0.5×10^6 / mL and a total volume of 1.5 mL and treated with the specified compound concentrations. Every 3 days, cell numbers were determined using a CASY model TT (Roche Innovatis) or a TC20 Automated Cell Counter (BioRad), the cells were then split back to the original seeding density if needed, and medium as well as compound were replaced. Based on microscopic inspection of the cultures, we had reason to believe that cell counts generated with the CASY instrument did not reliably exclude all dead cells. Therefore, percent of dead cells at the endpoint of the respective growth curves was measured by manually counting cells in a hemocytometer

using Trypan blue dye exclusion. Samples counted on a TC20 were always stained with Trypan blue, allowing the instrument to exclude dead cells.

Cell proliferation assay

For proliferation assays with thapsigargin, cells were pretreated during 3 days either with DMSO or 1 µM SGC0946 and subsequently seeded on 96-well plates in triplicate at 80 000 cells per well and incubated with various concentrations of thapsigargin for 24 h followed by quantification of viable cells using CellTiter-Glo (Promega). IC50 values were determined with the XLFit Excel Add-In (ID Business Solutions).

Genetic alterations in multiple myeloma (MM) cell lines

We compiled a list of frequently altered genes in MM from two sources: 11 genes highlighted in an analysis of paired tumor/normal samples from 203 MM patients {Lohr, 2014 #159}, and 22 genes from a comprehensive genomic characterization of 30 human MM cell lines {Vikova, 2019 #160}. Mutation data was downloaded from cBioPortal {Cerami, 2012 #162;Gao, 2013 #163} and mutation/translocation data on *WHSC1* (*NSD2*) was added {Jaffe, 2013 #161}. 15 genes were found to be altered in at least one cell line, for which we had determined sensitivity to DOT1L inhibition.

Histone methyltransferase panel of biochemical assays

IC50 values for SGC0946 and Compound 11 in a panel of 12 histone methyltransferase assays, including DOT1L, were determined for as described before {Qi, 2012 #158}.

In vivo experiment in a MM1-S mouse xenograft model

The experimental procedures involving animal studies strictly adhered to the Association for Assessment and Accreditation of Laboratory Animal Care International guidelines as published in the Guide for the Care and Use of Laboratory Animals, and to Novartis Corporate Animal Welfare policies. Studies were performed at Novartis facilities. Subcutaneous tumors were induced by injecting MM1-S cells bearing a luciferase reporter (MM1-S-luc) in HBSS containing 50% BD Matrigel in the flank of NOD-SCID mice (NOD/MrkBomTac-Prkdscid). Compound 11 was formulated as solution in 20% Solutol HS15/80% Saline and administered subcutaneously. Treatment was started when the average tumor size had reached approximately 150 mm^3 (n = 8/group). Tumor size was measured twice a week with a caliper. Tumor volume was calculated using the formula (Length × Width) × π/6 and expressed in mm^3. Data is presented as mean ± SEM. The statistical analysis was performed on delta tumor volume (ΔTVol) by comparing the treatment groups to the vehicle control group at endpoint by Kruskal-Wallis followed by Dunn's post-hoc test.

Quantitative real-time PCR

For mRNA expression analysis, total RNA was extracted from cells using an RNeasy mini kit (Qiagen, #74136) and reverse transcribed using a High Capacity cDNA Reverse Transcription Kit (Applied Biosystems, #4368814) according to the manufacturer's protocol. Real time PCR was then performed using Taqman Gene expression Master Mix (Applied Biosystems, #4369016). Expression was normalized to human 18S. The following predesigned labeled primers and probe sets (Taqman assays) from Life Technologies were used: hATF4 # HS.PT.561744435g; hASNS # HS.PT.561.28032225; hDDIT3 # HS.PT.58.34000360; hERN1 # HS.PT.58. 26321378; hHERPUD1 # HS. PT.58.21409911; h18S # HS. PT.39a.22214856g

Western blot analysis

After treatment with the indicated compounds, cells were collected, washed with 1xPBS and lysed in RIPA lysis buffer 10x (#20-188, Millipore), diluted in H_20 with PhosSTOP EASYpack (# 04906837001, Roche) and Complete Mini-Protease inhibitor cocktail tablets (11836153001, Roche). Samples were incubated on ice for 10 min, centrifuged 10 min at 10 000 rpm and then transferred into new precooled tubes. Protein concentration was assessed using a BSA kit (#23225, Pierce). Samples were then prepared in the following buffer: NuPAGE Sample Reducing Agent (10×, Invitrogen) diluted 1:10 with NuPAGE LDS Sample Buffer (4×, Invitrogen) and then boiled at 70° C for 10 min. Proteins were resolved by SDS-PAGE and transferred to a PVDF membrane using wet blotting system (Invitrogen). According to the experiment, the immunoblot was probed with the following antibodies: ATF4 (D4B8), rabbit mAb (CST, #11815); MYC, rabbit mAb (Abcam, #3ab2072; Actin, mouse mAb (Millipore Sigma, MAB1501); IRE1a (14C10), rabbit mAb (Cell Signaling Technology, #3294); phospho-IRE1a, rabbit pAb (Novus Biologicals, NB100-2323); phospho-PERK (Thr980) (16F8), rabbit mAb (Cell Signaling Technology #3179); secondary antibody: anti-rabbit, goat pAb to Rb-IgG (Abcam, #7090); secondary antibody: anti-mouse, sheep IgG HRP-linked (GE healthcare, #NA931V). For the histone blot, cells were collected, washed with 1xPBS and then lysed in 0.5 M HCl. Samples were vortexed thoroughly, placed overnight at 4° C and then centrifuged at 2 000 rpm for 10 min at 4° C. Supernatants were transferred into new tubes and neutralized with equal volume of NaHPO4 (0.5 M pH 11.3, containing 2mM DTT and 2x PIC (#P8340, 100x, Sigma)). The same procedure as the one described above was then performed. The following antibodies were used:

H3K79me2, rabbit mAb (Millipore, #04-835); H3K4me3, rabbit pAb (Millipore, #07-473); H3, rabbit pAb (CST, #9715); secondary antibody: anti-rabbit ,donkey IgG HRP-linked (GE healthcare, #NA934). Detection was performed with either the Dection kit SuperSignal West Femto Maximum Sensitivity Substrate (Thermo Fisher, #34096), or the SuperSignal West Dura Extented Duration Substrate (Thermo Fisher, #34075).

Antibody secretion in MM cells

Quantification of culture supernatant immunoglobulin light-chains content by ELISA assay

Nunc MaxiSorp 384-well plates (Thermo Scientific, #464718)) were coated with 20 µl/well capturing antigen diluted to 1 µg/ml in PBS (unlabeled goat anti-Kappa SouthernBiotech, #2060-01; unlabeled goat anti-Lambda SouthernBiotech, #2070-01). After overnight incubation at 4° C, plates were washed with PBS + 0.025% Tween-20 (Sigma-Aldrich) and 50 µl blocking solution (PBS + 0.5% bovine serum albumin (Sigma, # A9647)) were dispensed into each well. After 2 h of blocking at room temperature, plates were aspirated and prepared for sample dispensing. Liquid handling in the 384-well format was performed with a ViaFlo 384 unit (Integra Biosciences). Briefly, supernatants from MM cell lines cultures were transferred to a 384-deep well plate containing dilution buffer (PBS + 1% BSA). Serial dilutions were applied and 20 µl/well diluted supernatants were transferred to antigen-coated ELISA plates, incubated overnight at 4° C, then washed with 200 µl PBS + 0.025% Tween-20 (Sigma-Aldrich). Secondary antibodies of the appropriate isotype specificity (Goat anti-human Kappa and anti-human Lambda, HRP conjugated, SouthernBiotech #2060-05 and #2070-05, respectively) diluted in PBS + 0.5% BSA were added to each well at the appropriate volume and incubated 1 h at room temperature. Plates were finally washed with 250 µl/well PBS + 0.025% Tween-20 and aspirated before incubation with TMB soluble substrate (Seramun, Diagnostica GmbH), # S-001-2TMB) reaction termination with 0.25M H_2SO_4. Absorbance (OD405) of each well was measured using Gen5 2.01 software (BioTek). In the experiment shown in Supplementary Figure 3c, a Sebia FLC kit was used.

Quantification of immunoglobulin light-chains secreting plasma cells by ELISPOT assay

For immunoglobulin light chains secreting plasma cells quantification, 96-well ELISPOT plates (Millipore # MSIPS4510 Sterile, hydrophobic high protein binding immobilon-P membrane) were coated with 1 µg/ml purified unlabeled goat anti-Kappa (SouthernBiotech #2060-01); unlabeled goat anti-Lambda (SouthernBiotech # 2070-0) for 2 h at room temperature. After washing with PBS solution the plates were blocked with 1% BSA in PBS and incubated for 30 min at 37° C. Serial dilutions of cultured MM cell lines were added in a final volume of 200 µl of complete cell medium (RPMI1640 (Gibco, #11875093) 10% FBS (Hyclone)) and incubated overnight at 37° C. Next, the plates were washed 3 times with PBS 0.25% Tween20, 4 times with PBS and incubated 2 h at room temperature with immunoglobulin light chain-specific, biotin-conjugated secondary antibody (Southern Biotechnologies, #2060-08 and # 2070-08). After washing, avidin-peroxidase (horseradish peroxidase, HRP, Sigma-Aldrich, #A3151-1MG) was added and incubated for 1 h at room temperature. The assay was developed with AEC (Sigma-Aldrich, #A6926). For quantification of antibody secreting cells (ASCs), plates were acquired, counted (normalized to viable cells) and quality controlled using an ELISPOT reader and ImmunoSpot 5.1 software (CTL, Europe GmbH).

Viability assay and flow cytometry

Viability assay was performed by incubating the cells with Zombie Aqua Fixable Viability Kit (BioLegend, # 423101) 15 min at room temperature. For flow cytometer analysis, cells were incubated 20 min at 4° C with fluorochrome-conjugated mAbs specific for surface antigens and diluted in PBS containing 2% of FCS prior to analysis. The following monoclonal antibodies were used in this study: kappa APC (SouthernBiotech, #9180) and lambda PE (Southern Biotech, #9230-11). For intracellular staining, the cells were fixed and permeabilized using FIX & PERM Cell Fixation and Cell Permeabilization Kit (ThermoFisher Scientific, #GAS003)). Briefly, the cells were incubated 15 min at room temperature with Fixation medium. After a washing step, the cell were incubated with the Permeabilization medium and the recommended volume of intracellular antibodies (kappa- APC- Southern Biotech, #9180 and lambda-PE- Southern Biotech, #9230-11) for 20 min at room temperature. After washing, the cells were resuspended in PBS + 2% FCS for immediate analysis. Labeled cells were analyzed on a LSRFortessa (BD Biosciences) and the data were analyzed using FlowJo software.

REFERENCES

1. Barretina J, Caponigro G, Stransky N, Venkatesan K, Margolin AA, Kim S, Wilson CJ, Lehar J, Kryukov GV, Sonkin D, Reddy A, Liu M, Murray L, Berger MF, Monahan JE, Morais P, et al. The Cancer Cell Line Encyclopedia enables predictive modelling of anticancer drug sensitivity. Nature. 2012; 483:603–607. https://doi.org/10.1038/nature11003. [PubMed]
2. Yu W, Chory EJ, Wernimont AK, Tempel W, Scopton A, Federation A, Marineau JJ, Qi J, Barsyte-Lovejoy D, Yi J, Marcellus R, Iacob RE, Engen JR, Griffin C, Aman A, Wienholds E, et al. Catalytic site remodelling of the DOT1L methyltransferase by selective inhibitors. Nat Commun. 2012; 3:1288. https://doi.org/10.1038/ncomms2304. [PubMed]

3. Schuierer S, Roma G. The exon quantification pipeline (EQP): a comprehensive approach to the quantification of gene, exon and junction expression from RNA-seq data. Nucleic Acids Res. 2016; 44:e132. https://doi.org/10.1093/nar/gkw538. [PubMed]
4. Law CW, Chen Y, Shi W, Smyth GK. voom: Precision weights unlock linear model analysis tools for RNA-seq read counts. Genome Biol. 2014; 15:R29. https://doi.org/10.1186/gb-2014-15-2-r29. [PubMed]
5. Ritchie ME, Phipson B, Wu D, Hu Y, Law CW, Shi W, Smyth GK. limma powers differential expression analyses for RNA-sequencing and microarray studies. Nucleic Acids Res. 2015; 43:e47. https://doi.org/10.1093/nar/gkv007. [PubMed]
6. Potting C, Crochemore C, Moretti F, Nigsch F, Schmidt I, Manneville C, Carbone W, Knehr J, DeJesus R, Lindeman A, Maher R, Russ C, McAllister G, Reece-Hoyes JS, Hoffman GR, Roma G, et al. Genome-wide CRISPR screen for PARKIN regulators reveals transcriptional repression as a determinant of mitophagy. Proc Natl Acad Sci U S A. 2018; 115:E180–E189. https://doi.org/10.1073/pnas.1711023115. [PubMed]
7. Subramanian A, Tamayo P, Mootha VK, Mukherjee S, Ebert BL, Gillette MA, Paulovich A, Pomeroy SL, Golub TR, Lander ES, Mesirov JP. Gene set enrichment analysis: a knowledge-based approach for interpreting genome-wide expression profiles. Proc Natl Acad Sci U S A. 2005; 102:15545–15550. https://doi.org/10.1073/pnas.0506580102. [PubMed]
8. Balwierz PJ, Pachkov M, Arnold P, Gruber AJ, Zavolan M, van Nimwegen E. ISMARA: automated modeling of genomic signals as a democracy of regulatory motifs. Genome Res. 2014; 24:869–884. https://doi.org/10.1101/gr.169508.113. [PubMed]
9. Orlando DA, Chen MW, Brown VE, Solanki S, Choi YJ, Olson ER, Fritz CC, Bradner JE, Guenther MG. Quantitative ChIP-Seq normalization reveals global modulation of the epigenome. Cell Rep. 2014; 9:1163–1170. https://doi.org/10.1016/j.celrep.2014.10.018. [PubMed]

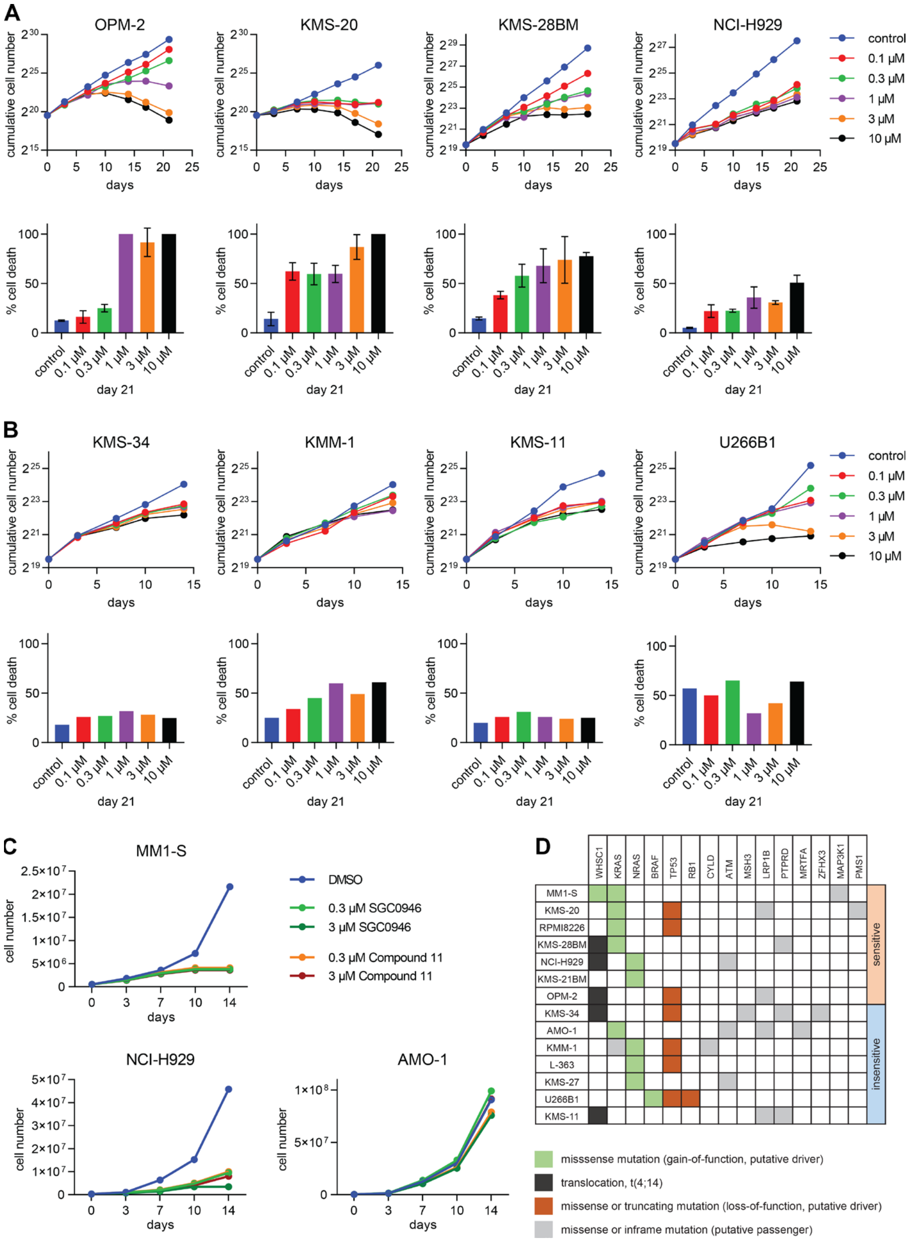

Supplementary Figure 1: (**A**) The effect of the DOT1L inhibitor SGC0946 on the growth and viability of the indicated MM cell lines, measured by CASY TT cell counter (cumulative cell number) and at endpoint by manual counting in a hemocytometer with Trypan Blue (% cell death, mean ± s.d.) as in Figure 1A. (**B**) Cumulative cell numbers and % cell death in the indicated MM cell lines measured with a T20 cell counter in presence of Trypan Blue. (**C**) The effect of two different DOT1L inhibitors, Compound 11 and SGC0946, on the growth of the 3 indicated cell lines (T20 cell counter). (**D**) Matrix displaying genetic alterations of 15 genes that are frequently altered in MM across 14 MM cell lines for which we had determined sensitivity to DOT1L inhibition. *KRAS* gain-of-function mutations showed the most imbalanced distribution between sensitive and insensitive cell lines, but this difference was not statistically significant (Fisher's exact test, *P*-value = 0.2657).

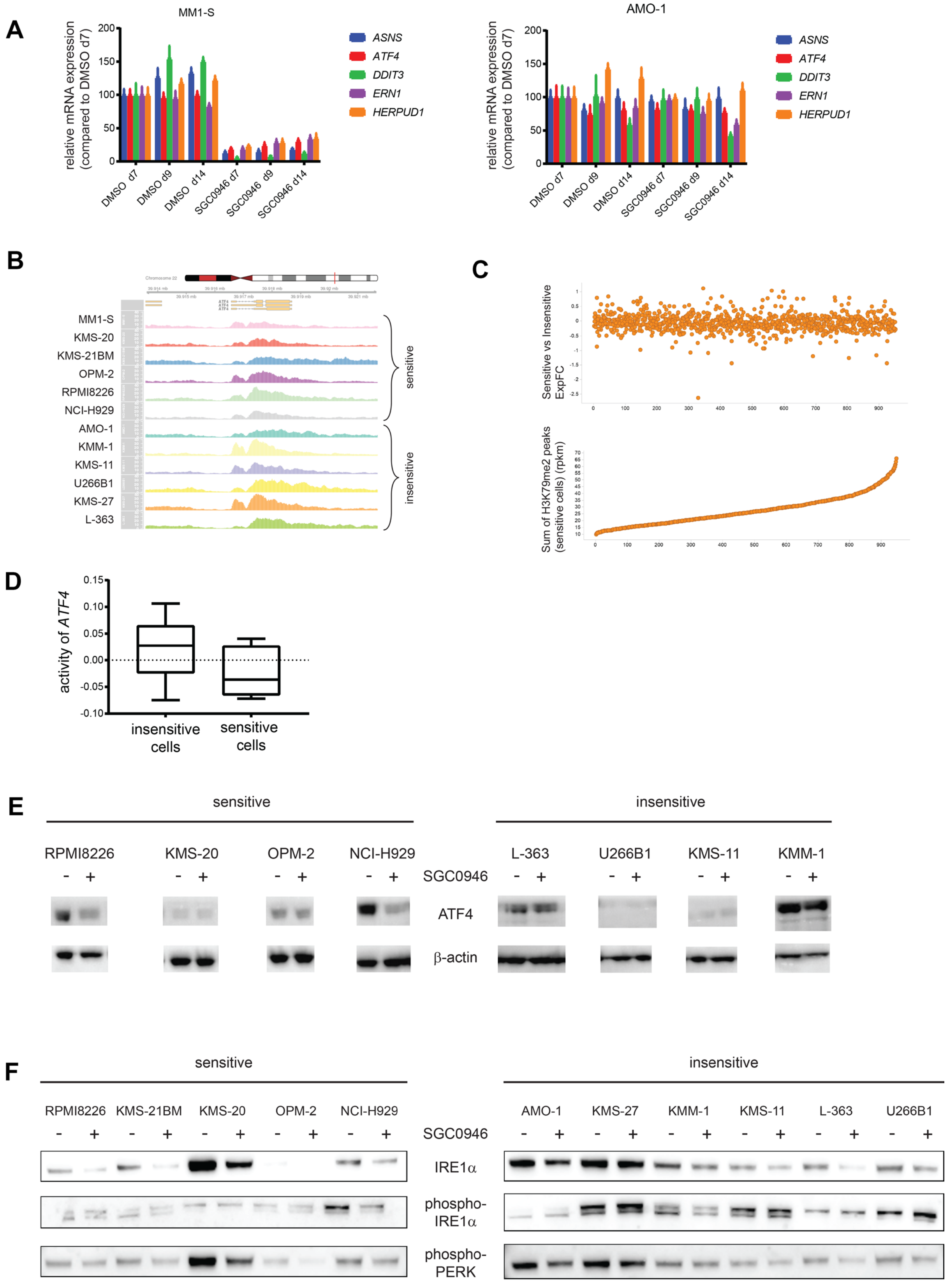
A
MM1-S
AMO-1
relative mRNA expression (compared to DMSO d7)
ASNS
ATF4
DDIT3
ERN1
HERPUD1
DMSO d7
DMSO d9
DMSO d14
SGC0946 d7
SGC0946 d9
SGC0946 d14
B
MM1-S
KMS-20
KMS-21BM
OPM-2
RPMI8226
NCI-H929
AMO-1
KMM-1
KMS-11
U266B1
KMS-27
L-363
sensitive
insensitive
C
Sensitive vs Insensitive ExpFC
Sum of H3K79me2 peaks (sensitive cells) (rpkm)
D
activity of ATF4
insensitive cells
sensitive cells
E
sensitive
insensitive
RPMI8226
KMS-20
OPM-2
NCI-H929
SGC0946
L-363
U266B1
KMS-11
KMM-1
ATF4
β-actin
F
sensitive
insensitive
RPMI8226
KMS-21BM
KMS-20
OPM-2
NCI-H929
SGC0946
AMO-1
KMS-27
KMM-1
KMS-11
L-363
U266B1
IRE1α
phospho-IRE1α
phospho-PERK

G

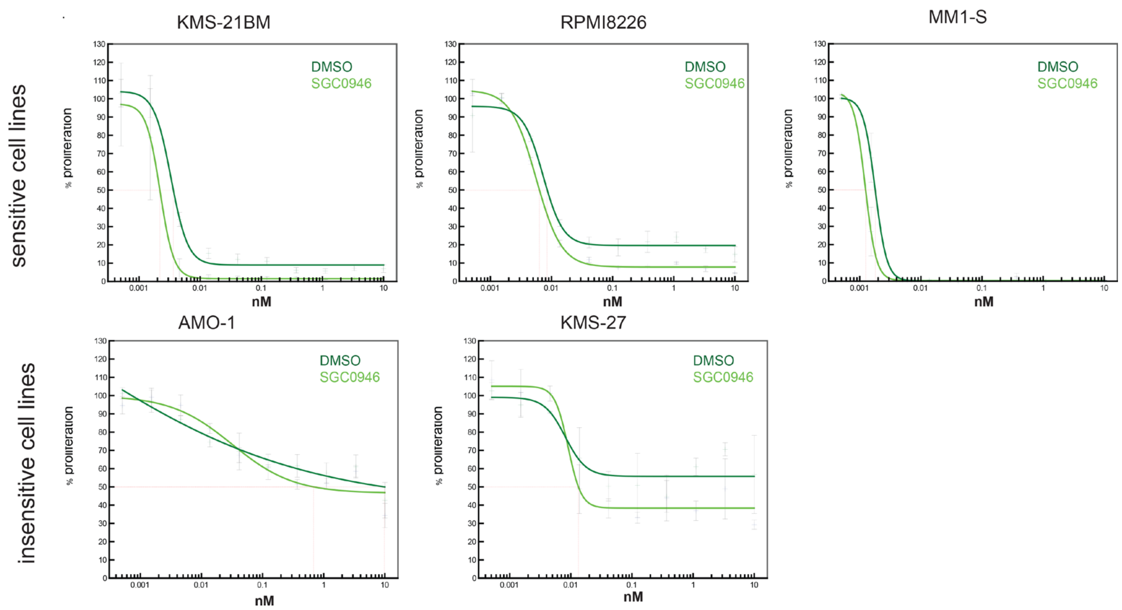

Supplementary Figure 2: (**A**) Validation of expression changes of 5 different transcripts (*ASNS*, *ATF4*, *DDIT3*, *ERN1*, *HERPUD1*) in MM1-S (sensitive cell line) and AMO-1 (insensitive cell line) by reverse transcriptase quantitative real-time PCR (RT-qPCR). Cells were treated with 1 µM SGC0946 for 14 days and RNA was collected at different time points (day 7, day 9 and day 14). (**B**) ChIP-seq tracks representing signal for H3K79me2 in the MM cell lines around the *ATF4* gene. (**C**) Plots correlating gene expression changes upon DOT1L inhibition and intensity of H3K79me2 profiles. Expression log2-fold change between sensitive and insensitive cells as in Figure 2B (upper panel). Genes are ranked by intensity of H3K79me2 peaks (sum of sensitive cell lines; lower panel) rpkm: reads per kilobase million. (**D**) Activity of the TF ATF4 at basal level in the sensitive and insensitive cell lines. Difference not significant (P-value > 0.05). (**E**) ATF4 protein levels as in Figure 2F, but in additional cell lines. (**F**) Protein levels of IRE1α, phospho-IRE1α (S274), and phospho-PERK (T980) in the indicated cell lines after 6 days of treatment with either 1 µM SGC0946 or DMSO as control. (**G**) Cell proliferation assay of KMS-21BM, RPMI8226, MM1-S (sensitive cell lines) and AMO-1, KMS-27 (insensitive cell lines), treated for 24 h with thapsigargin (curve fit using 4-parameter dose-response model from XLFit).

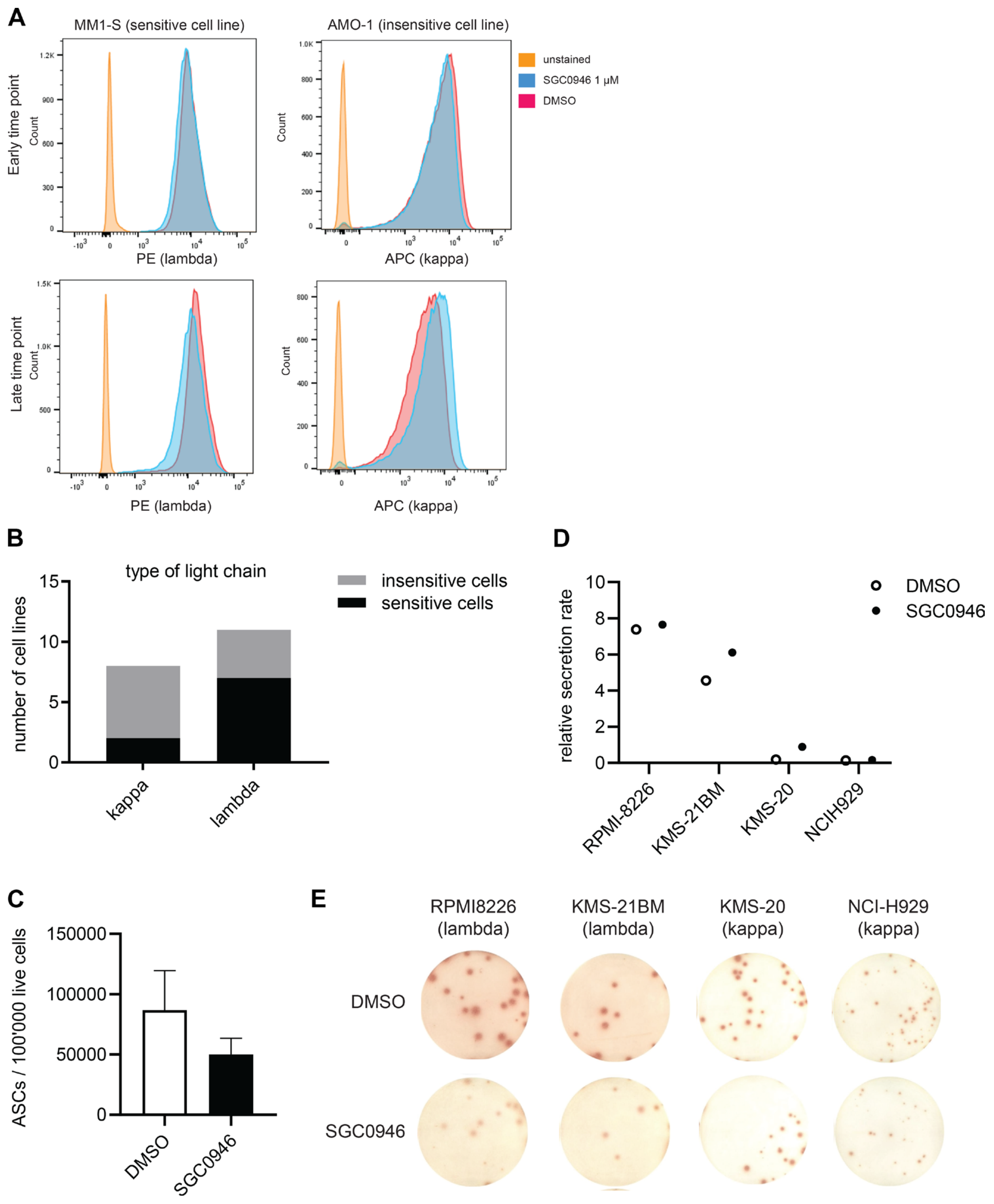

Supplementary Figure 3: (**A**) Cell surface lambda or kappa light chains detected by flow cytometry in MM1-S and AMO-1 cells during incubation with 1 µM SGC0946 or DMSO at an early time point (3 days) and late time point (6 days). Data for other cell lines not shown. (**B**) Graph representing the number of sensitive or insensitive cell lines producing either kappa or lambda light chains (left panel). A trend towards association of sensitivity with lambda light chain production was observed (Fisher's exact test, *P*-value < 0.17). (**C**) Sensitive MM cell lines of lambda (RPMI-8226, KMS-21BM) and kappa (KMS-20, NCI-H929) isotype were treated in vitro with 1 µM SGC0946 or DMSO as vehicle control. At day 6, cells were recovered, washed 4 times and seeded into an ELISPOT plate to quantify the number of antibody secreting cells (ASCs). An average ± s.d. of the 4 tested cell lines is shown. (**D**) MM cell lines were treated as in (C). At day 6, secreted light chain antibodies in the culture supernatant from all tested conditions were quantified, using an ELISA assay and human-kappa and -lambda as standards. To determine antibody secretion rates, the amount of antibodies quantified in 200 µl of culture supernatant was normalized on the total number of antibody secreting cells (ASCs) determined by ELISPOT in each condition. (**E**) Representative images from the ELISPOT assay showing that the size of the spots generated by ASCs treated with SGC0946 or DMSO.

A

NAME
KRIGE_AMINO_ACID_DEPRIVATION
MORI_PLASMA_CELL_UP
BLUM_RESPONSE_TO_SALIRASIB_UP
GO_CELLULAR_RESPONSE_TO_TOPOLOGICALLY_INCORRECT_PROTEIN
PENG_LEUCINE_DEPRIVATION_UP
GSE29164_CD8_TCELL_VS_CD8_TCELL_AND_IL12_TREATED_MELANOMA_DAY7_DN
SHAFFER_IRF4_TARGETS_IN_PLASMA_CELL_VS_MATURE_B_LYMPHOCYTE
BHATI_G2M_ARREST_BY_2METHOXYESTRADIOL_DN
ZHAN_MULTIPLE_MYELOMA_CD1_VS_CD2_UP
REACTOME_TRANSLATION
REACTOME_SRP_DEPENDENT_COTRANSLATIONAL_PROTEIN_TARGETING_TO_MEMBRANE
GO_IRE1_MEDIATED_UNFOLDED_PROTEIN_RESPONSE
HELLER_HDAC_TARGETS_SILENCED_BY_METHYLATION_DN
GSE29614_DAY3_VS_DAY7_TIV_FLU_VACCINE_PBMC_DN
SHAFFER_IRF4_TARGETS_IN_MYELOMA_VS_MATURE_B_LYMPHOCYTE
GO_PROTEIN_LOCALIZATION_TO_ENDOPLASMIC_RETICULUM
GO_ESTABLISHMENT_OF_PROTEIN_LOCALIZATION_TO_ENDOPLASMIC_RETICULUM
GO_RESPONSE_TO_ENDOPLASMIC_RETICULUM_STRESS
HALLMARK_UNFOLDED_PROTEIN_RESPONSE
REACTOME_3_UTR_MEDIATED_TRANSLATIONAL_REGULATION
GSE2770_UNTREATED_VS_IL12_TREATED_ACT_CD4_TCELL_6H_UP
ALK_DN.V1_UP
GO_TRANSLATIONAL_INITIATION
SHAFFER_IRF4_TARGETS_IN_ACTIVATED_DENDRITIC_CELL
GSE29614_CTRL_VS_DAY7_TIV_FLU_VACCINE_PBMC_DN
GO_PROTEIN_N_LINKED_GLYCOSYLATION
SHAFFER_IRF4_MULTIPLE_MYELOMA_PROGRAM
TARTE_PLASMA_CELL_VS_B_LYMPHOCYTE_UP
GO_NUCLEAR_TRANSCRIBED_MRNA_CATABOLIC_PROCESS_NONSENSE_MEDIATED_DECAY
REACTOME_NONSENSE_MEDIATED_DECAY_ENHANCED_BY_THE_EXON_JUNCTION_COMPLEX
GO_PEPTIDYL_ASPARAGINE_MODIFICATION
HELLER_HDAC_TARGETS_DN
PACHER_TARGETS_OF_IGF1_AND_IGF2_UP
REACTOME_PEPTIDE_CHAIN_ELONGATION
GNF2_EIF3S6
TIEN_INTESTINE_PROBIOTICS_24HR_DN
KEGG_RIBOSOME
ZHAN_MULTIPLE_MYELOMA_CD1_UP
GNF2_ST13
REACTOME_UNFOLDED_PROTEIN_RESPONSE

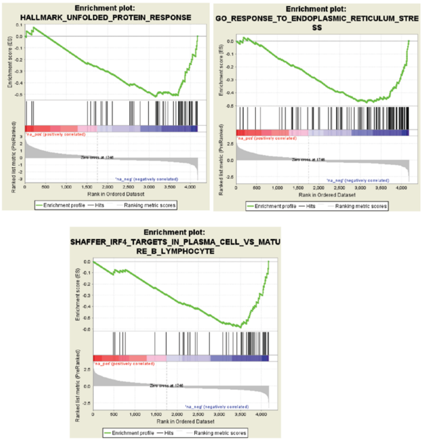

B

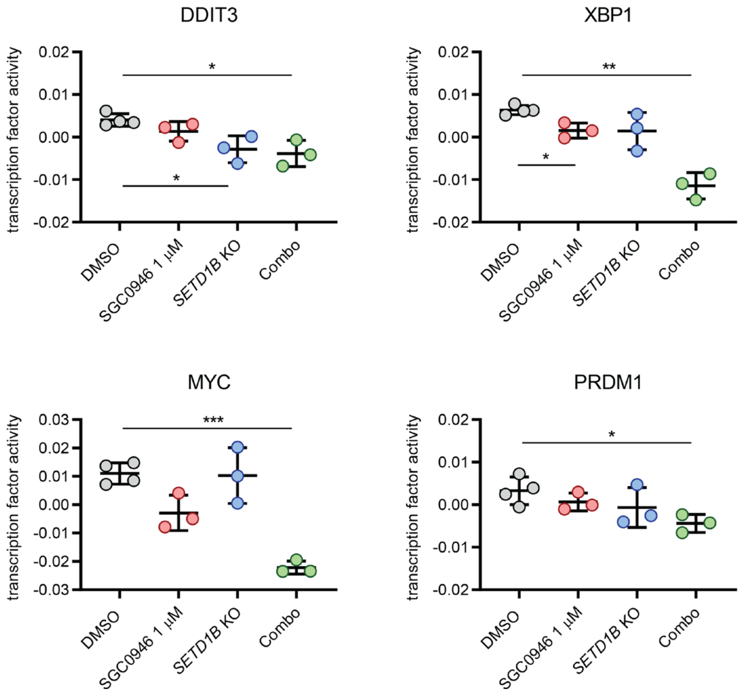

Supplementary Figure 4: (**A**) GSEA analysis of the RNA-seq data, (downregulated genes when comparing combined DOT1L inhibition and *SETD1B* targeting by CRISPR versus DMSO), related to Figure 5. (**B)** Activity of the indicated TFs by ISMARA as in Figure 5D. All *P*-values were obtained from unpaired two-sided *t*-tests: ****P*-value < 0.001, ***P*-value < 0.01, **P*-value < 0.05.

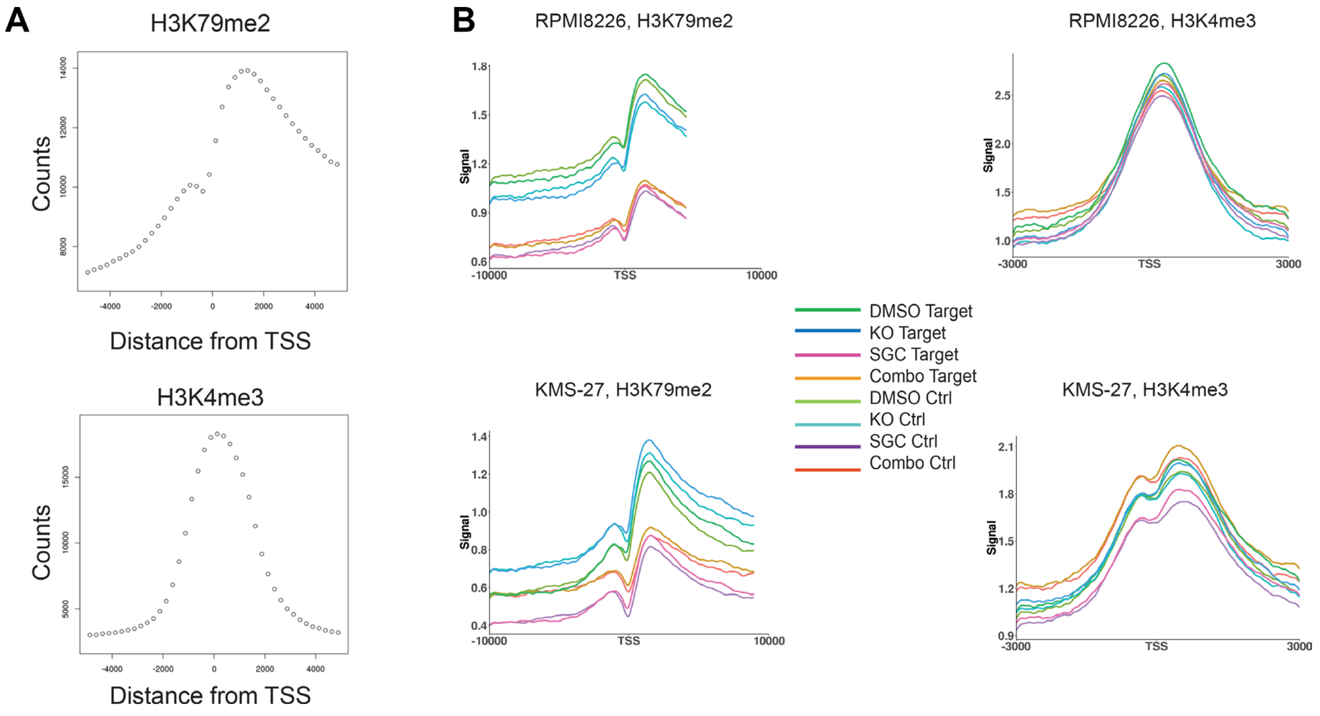

A
H3K79me2
Counts
Distance from TSS
H3K4me3
Counts
Distance from TSS
B
RPMI8226, H3K79me2
RPMI8226, H3K4me3
KMS-27, H3K79me2
KMS-27, H3K4me3
Signal
TSS
DMSO Target
KO Target
SGC Target
Combo Target
DMSO Ctrl
KO Ctrl
SGC Ctrl
Combo Ctrl

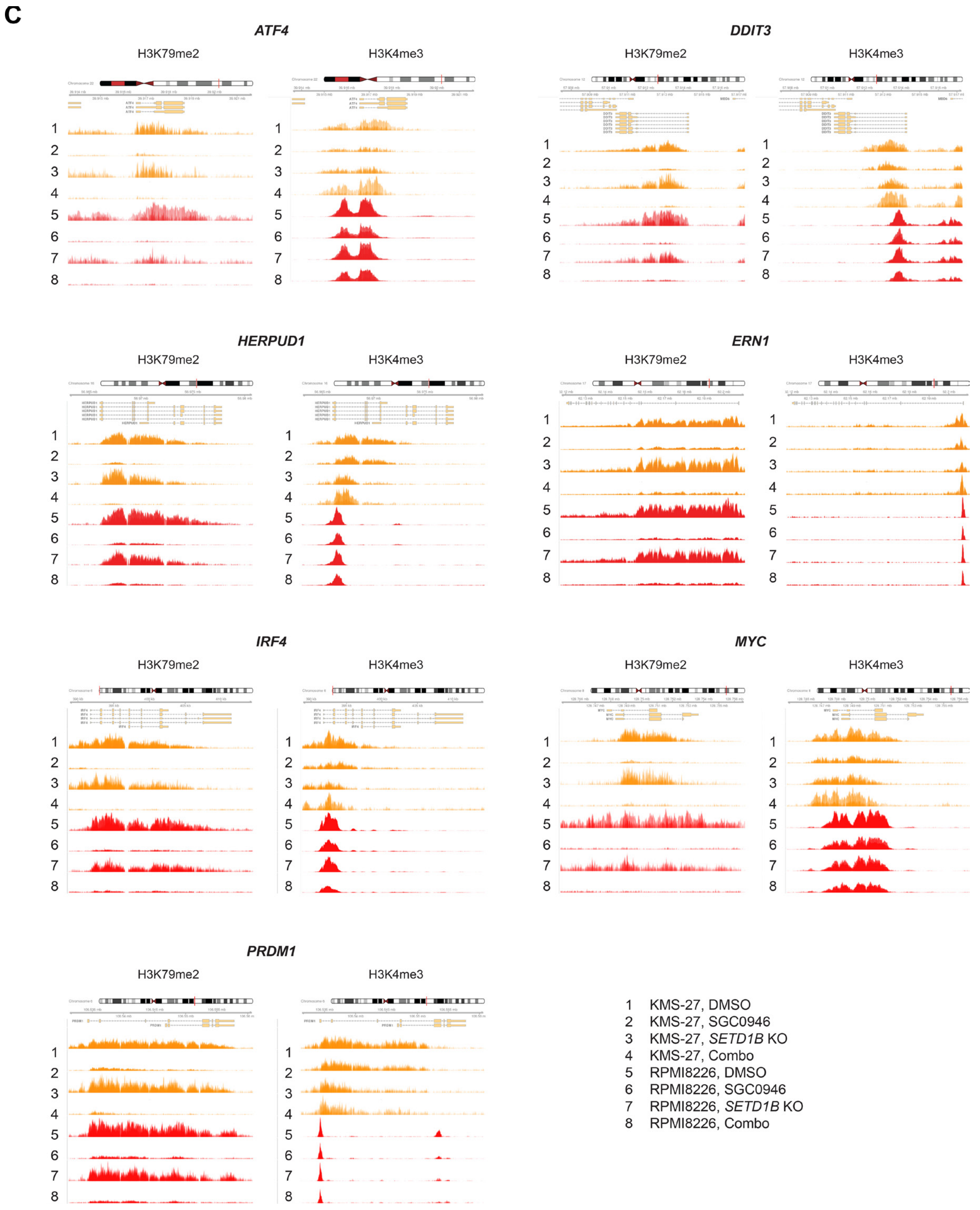

Supplementary Figure 5: (**A**) Aggregated ChiP-seq signal at TSS for H3K79me2 and H3K4me3. (**B**) H3K79me2 and H3K4me3 metagene profiles around the TSS of ATF4 target genes (Target) and control genes (Ctrl) in RPMI8226 and KMS-27 Cas9 cells with inducible sgSETD1B, treated with either DMSO (dark green target genes, light green control genes), 1 µM SGC0946 (SGC, red target genes, yellow control genes), 100 ng/ml dox (KO, blue target genes, brown control genes), or a combination of SGC0946 and dox (Combo, violet target genes, grey control genes). (**C**) ChIP-seq tracks representing signal for H3K79me2 and H3K4me3 around *ATF4*, *DDIT3*, *HERPUD1*, *ERN1*, *IRF4*, *MYC*, and *PRDM1* genes in RPMI8226 and KMS-27 Cas9 (with inducible sgSETD1B), treated as in B.

Supplementary Table 1: Potency and selectivity of SGC0946

	**SGC0946**
SmyD2	>10
Set7/9	>10
SetD8	>10
Ezh2	>10
Ezh1	>10
MLL	>10
Suv39H2	>10
G9a	>10
SETD2	>10
NSD3	>10
Dot1L	0.01
CARM1	>10

IC50 values (mM) for the DOT1L inhibitor SGC0946 tested across a panel of 12 histone methyltransferase (HMT) biochemical assays.
Details on the HMT panel are published in Qi W, Chan H, Teng L, et al. Proc Natl Acad Sci U S A. 2012 Dec 26;109:21360–5.

Supplementary Table 2: Transcription factor activity changes upon DOT1L inhibition (ISMARA analysis) that distinguish sensitive and insensitive cell lines

Transcription factor(s)	*P*-value	difference
ATF4	0.0050	-1.06
CEBPB	0.0045	-0.73
ETV1_ERF_FEV_ELF1	0.0009	-0.55
ELK4_ETV5_ELK1_ELK3_ELF4	0.0018	-0.48
RELB	0.0272	-0.42
SREBF1_TFE3	0.0143	-0.38
DDIT3	0.0058	-0.36
RFX7_RFX4_RFX1	0.0048	-0.35
BACH2	0.0426	-0.34
ATF6	0.0148	-0.34
ZNF282	0.0141	-0.34
SIX5_SMARCC2_HCFC1	0.0063	-0.33
PRDM1	0.0265	-0.33
MZF1	0.0497	-0.32
SOX13_SOX12	0.0428	-0.32
RXRG	0.0324	-0.31
NKX6.2	0.0072	-0.29
HLF_TEF	0.0087	-0.29
PKNOX1_TGIF2	0.0475	-0.27
NR4A1	0.0419	-0.26
BBX	0.0009	-0.26
ETV6	0.0409	-0.25
RORA	0.0464	-0.21
GCM1	0.0425	-0.21
DRGX_PROP1	0.0018	-0.20
HDX	0.0227	-0.19
PAX1_PAX9	0.0426	-0.19
OLIG3_NEUROD2_NEUROG2	0.0475	-0.18
ZBTB7A_ZBTB7C	0.0489	-0.18
ZNF711_TFAP2A_TFAP2D	0.0466	-0.14
DLX5	0.0251	-0.13
PAX5	0.0345	-0.11
SPIB	0.0289	0.87
SPIC	0.0095	0.66
FOXA3_FOXC2	0.0006	0.55
HIC1	0.0027	0.54
MEF2C	0.0180	0.45
ERG	0.0089	0.44
ID4_TCF4_SNAI2	0.0275	0.36

MAFF_MAFG	0.0176	0.34
NKX3.1	0.0001	0.32
EZH2	0.0388	0.27
MEF2B	0.0476	0.25
ZNF263	0.0434	0.24
WT1_MTF1_ZBTB7B	0.0232	0.21
POU4F2	0.0403	0.19
NFIX_NFIB	0.0304	0.17
E4F1	0.0461	0.14
NKX6.3	0.0215	0.13
TBX4	0.0258	0.10
ZBED1	0.0425	0.09

The table lists transcription factors, whose change in activity (ISMARA score) upon DOT1L inhibition with SGC0946 as significantly different between sensitive and insensitive cell lines.
P-values are from t-tests comparing the SGC0946-induced changes in sensitive vs insensitive cell lines, filtered for $P < 0.05$.
Difference = (average change in insensitive cell lines) - (average change in sensitive cell lines).

Supplementary Table 3: Gene expression differences between sensitive and insensitive MM cell lines at baseline. See Supplementary Table 3

Supplementary Table 4: Gene expression changes in RPMI8226 cells upon DOT1L inhibition, SETD1B knockout, or combination thereof after 3 days. See Supplementary Table 4
